# Chemically Selective Alternatives to Photoferroelectrics for Polarization‐Enhanced Photocatalysis: The Untapped Potential of Hybrid Inorganic Nanotubes

**DOI:** 10.1002/advs.201600153

**Published:** 2016-09-13

**Authors:** Joshua D. Elliott, Emiliano Poli, Ivan Scivetti, Laura E. Ratcliff, Lampros Andrinopoulos, Jacek Dziedzic, Nicholas D. M. Hine, Arash A. Mostofi, Chris‐Kriton Skylaris, Peter D. Haynes, Gilberto Teobaldi

**Affiliations:** ^1^ Stephenson Institute for Renewable Energy and Department of ChemistryUniversity of LiverpoolLiverpoolL69 3BXUK; ^2^ The Thomas Young Centre for Theory and Simulation of MaterialsImperial College LondonLondonSW7 2AZUK; ^3^ School of ChemistryUniversity of SouthamptonSouthamptonSO17 1BJUK; ^4^ Faculty of Applied Physics and MathematicsGdansk University of TechnologyGdansk80 233Poland; ^5^ Department of PhysicsUniversity of WarwickCoventryCV4 7ALUK

**Keywords:** chemical separation, ferroelectrics, hybrid inorganic nanotubes, linear‐scaling density functional theory, photocatalysis

## Abstract

**Linear‐scaling density functional theory simulation of methylated imogolite nanotubes (NTs)** elucidates the interplay between wall‐polarization, bands separation, charge‐transfer excitation, and tunable electrostatics inside and outside the NT‐cavity. The results suggest that integration of polarization‐enhanced selective photocatalysis and chemical separation into one overall dipole‐free material should be possible. Strategies are proposed to increase the NT polarization for maximally enhanced electron–hole separation.

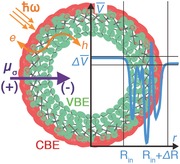

Photocatalytic materials (photocatalysts, PCs) are receiving increasing attention since they can exploit solar light energy for chemical fuels production,^1^ environmental remediation,^2^ or to access alternative, highly selective, excited‐state reaction paths for high‐value chemicals production.^3^ The basic requirements of good (visible) light‐absorbance, efficient separation of photogenerated electron–hole (e–h) pairs, independent e (h) diffusion to the PC‐surfaces and transfer to (different or selected) reactants, are clearly established.^1^, ^2^, ^3^ However, the fulfillment of such requirements by cheap and scalable materials remains elusive due to the poorly understood relationships between the properties of a PC and its atomic composition, structure, and solvent‐dependent interactions with reactants. Aiming at efficient e–h separation and diffusion to reactants, both 1D structuring of PCs^4^ and use of permanently polarized photoferroelectrics^5^ have started to be investigated and found to increase photocatalytic performance. Confinement of reactants and intermediates inside nanoporous PCs has also started to be explored and observed to benefit reaction selectivity.^3^ To foster optimized integration of these currently disconnected research strategies into chemically selective alternatives to photoferroelectrics for polarization‐enhanced photocatalysis, here we investigate an emerging class of cheap 1D nanomaterials, namely, hybrid inorganic–organic imogolite nanotubes (Imo‐NTs).^6^ Linear‐scaling density functional theory (DFT) simulations elucidate the interplay between NT functionalization, curvature, permanent polarization, band gap (BG), band‐separation, band‐alignment, and charge‐transfer excitations, enabling informed design of a novel class of locally polarized, selective porous PCs. Finally, we suggest strategies based on the synthetic flexibility of Imo‐NTs to increase the NT‐polarization for maximally enhanced electron–hole separation and photocatalytic reactivity.

Aluminosilicate (AlSi) and aluminogermanate (AlGe) Imo‐NTs are structurally analogous to the naturally occurring hydrous‐aluminosilicate imogolite.^7^ Their walls consist of a single layer of octahedrally coordinated aluminum hydroxide with pendant tetrahedral silanol (Si–OH) groups facing the tube cavity. From a compositional point of view, the only difference between AlSi and AlGe NTs is the substitution of silanol groups with germanol (Ge–OH) groups.

Synthetic control has grown noticeably, with the definition of synthetic routes to single‐walled AlSi and AlGe NTs of controllable radius and length^8^ as well as double‐walled AlGe NTs.^9^ Progress has also been made in the post‐synthetic selective functionalization of the outer or inner surface of AlSi NTs,^10^ and in the synthesis of hybrid organic–inorganic methylated (AlSi‐Me) or aminated AlSi NTs derivatives, that have —CH_3_ (**Figure** **1**a) and —CH_2_—NH_2_ groups in the NT cavity.^6^ Synthesis of hybrid methylated Al(Si/Ge)‐Me NTs with a tunable Si/Ge ratio has also been reported.[[qv: 6c]] The occurrence of a hydrophobic cavity inside an otherwise hydrophilic NT leads to superior chemical separation performances,^6^, ^10^ defining a potentially advantageous starting point for the integration of chemical separation and photocatalytic strategies in this class of porous 1D material.

**Figure 1:**
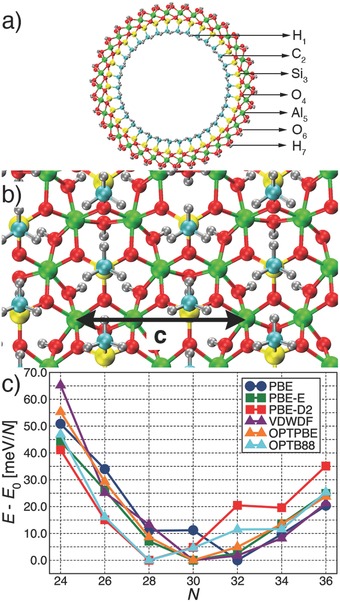
Front a) and side‐view from inside the NT‐cavity b) of the AlSi_34_‐Me NT structure. The black arrow marks the length of the repeat unit along the NT‐axis (c). Al: green, Si: yellow, C: cyan, O: red, H: gray. c) Relative DFT‐energy, normalized to the number of Al‐atoms in the NT (*N*) and referenced to the computed minimum, for each of the XC‐functional used.

The permanent polarization of the NT‐wall and a real‐space separation of the valence (VBE) and conduction band (CBE) edges of pristine AlSi and AlGe Imo‐NTs,^11^ suggest enhanced e–h separation, and selective reduction and oxidation of different reactants on different sides of the NT‐cavity, leading to integration of photocatalytic “Z‐schemes”^12^ across the NT‐wall. Crucially, and motivating this work, the changes to the Imo‐NTs' curvature, wall‐polarization, VBE–CBE separation, band‐alignment, and optical properties due to organic functionalization are to date unknown.

Here, we focus on methylated AlSi‐Me Imo‐NTs.[[qv: 6a–c]] As shown in Figure 1a,b, they present a hydrophilic outer surface and a hydrophobic cavity, which can very effectively separate hydrophobic and hydrophilic species.[[qv: 6a–c]] In spite of results on the diameter from N_2_ adsorption,[[qv: 6a]] powder X‐ray diffraction,[[qv: 6a]] and small‐angle X‐ray scattering (SAXS),[[qv: 6c]] to date no X‐ray quantitative resolution of the atomic structure of AlSi‐Me NTs is available, inviting simulation of the dependence of the system energy on the NT composition and structure. Given the occurrence of polarizable methyl groups in the NT‐cavity and, to the best of our knowledge, unavailability of previous benchmarks on the matter, in our study we considered six different exchange‐correlation (XC) functionals to assess the actual need of including dispersion corrections in the DFT modeling of organically functionalized metal‐hydroxides.

Geometry optimization of AlSi‐Me NTs with 24 to 36 Al‐atoms (*N*) in the circumference uncovers a broad and relatively shallow energy‐minimum between *N* = 28 (AlSi_28_‐Me from now on) and *N* = 34 (AlSi_34_‐Me), regardless of the XC‐functional used (Figure 1c). The inner (H_1_) and outer (H_7_) diameters for the optimized AlSi_28_‐Me and AlSi_34_‐Me NTs (H_1_:15.33–20.11 Å, H_7_: 26.33–31.07 Å, see Table S1 in the Supporting Information) bracket the experimental pore size distribution from N_2_ adsorption (peaked at ≈20 Å[[qv: 6a]]) and SAXS fitting (18.2 Å[[qv: 6c]]), confirming that AlSi‐Me NTs have diameter larger than pristine AlSi NTs (SAXS diameter: 14.8 Å[[qv: 6c]]). Despite changes in the fine‐details of the *E*(*N*) profile, which indicate a (likely medium dependent^8^) balance between covalent bonding, structural strain, outer hydrogen bonding, and dispersion interactions (Figure S5, Supporting Information) for the NTs' energy, the optimized NTs diameter and bond‐lengths are negligibly (±0.01 Å) affected by the XC‐functional (Tables S1 and S2, Supporting Information): covalent bonding of the aluminum hydroxide layer dominates over dispersion interactions for the structuring of the methylated NTs. This conclusion is corroborated by the negligible deviations (≤0.01 Å) between the bond‐lengths in the aluminum hydroxide layers of the pristine AlSi_24_ and AlSi_24_‐Me NTs (Table S2, Supporting Information). However, dispersion terms do affect the relative energy of the Imo unit for NTs of different diameters. In this respect, quantitative resolution of the atomic structure of the NTs, to the best of our knowledge currently not available, would be necessary to assess directly the accuracy of the XC‐functionals used for the considered NTs.

Appropriate alignment between the electronic bands of a PC and the e (h) acceptor states of reactants is critical for viable e (h) transfer and possible photoreduction (oxidation) chemistry. Although interface structuring and charge redistribution can greatly affect the electronic alignment between PC and reactants,^1^, ^2^ the position of the PC band edges with respect to the vacuum level can be used as a first approximation to the PC photoreduction(oxidation) energy drive, especially if compared with results for known PCs.

**Figure** **2**a shows the vacuum‐aligned VBE and CBE for the considered AlSi_N_‐Me NTs. Within deviations of 0.09 eV or less, the computed VBE and CBE for the AlSi_N_‐Me NTs, and the corresponding energy drive toward photoreduction (oxidation), are found to depend weakly on the NT diameter and curvature. Despite negligible effects on the optimized geometry (Tables S1 and S2, Supporting Information), explicitly nonlocal dispersion XC‐functionals (VDWDF, OPTPBE, OPTB88) yield band edges downshifted by roughly 0.2–0.3 eV with respect to the results of the (empirically corrected) semilocal PBE functional (Table S3, Supporting Information). The minimal (≤0.08 eV) deviations between PBE VBEs (CBEs) and the PBE‐results on the geometries optimized with non‐local dispersion XC‐functionals (Table S4, Supporting Information) indicate that the deviations between PBE and nonlocal dispersion XC‐functionals in Table S3 of the Supporting Information stem primarily from the different exchange treatment.

**Figure 2:**
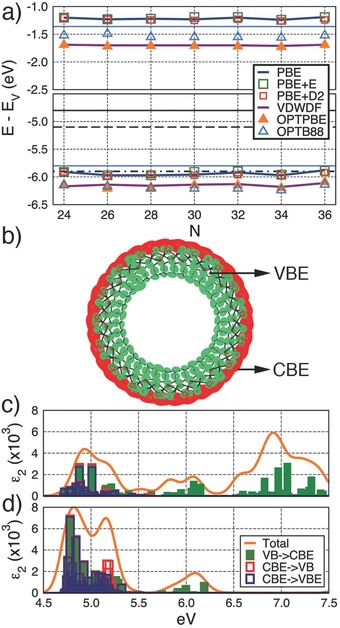
a) Vacuum‐aligned VBE and CBE of the AlSi_N_‐Me NTs for the range of adopted XC‐functionals. The experiment and hybrid meta‐GGA derived CBE of bulk anatase (−5.1 eV, dashed) and rutile (−4.8 eV, continuous) TiO_2_ (from ref. [[qv: 15a]]), are marked with black horizontal lines (hybrid meta‐GGA rutile VBE: −7.38 eV, anatase VBE: −8.30 eV [from ref. [[qv: 15a]]). PBE VBE (−7.4 eV) and CBE (−5.9 eV) values for a vacuum‐exposed three‐layer rutile TiO_2_(110) slab[[qv: 15a]] are marked by dotted‐dashed lines. The VBE and CBE of the pristine AlSi_24_ Imo‐NT (PBE) are marked by the blue solid horizontal line. b) Real‐space separation between the VBE (green) and CBE (red) of AlSi_34_‐Me. The PBE imaginary component of the dielectric function (ε_2_) with single‐electron transition‐resolved analysis for (c) AlSi_28_‐Me and d) AlSi_34_‐Me. The VBE and CBE are defined as the top and bottom 0.5 eV of the VB and CB, respectively. Transitions from the VBE (whole VB) to the whole CB (CBE) are labeled as “VBE→CB” (“VB→CBE”). Transition between VBE and CBE are marked as “VBE→CBE”.

At the PBE level, the computed BG for the considered AlSi_N_‐Me NTs (4.70–4.75 eV depending on *N*, Table S3, Supporting Information) is 0.31–0.26 eV larger than for the pristine AlSi (*N* = 24) system (4.4 eV). Thus, methylation of the AlSi NT cavity increases the system BG. A non‐negligible, weakly diameter dependent, transfer of roughly 0.3 e/CH_3_ from the aluminum hydroxide backbone to the (negatively charged) methyl groups accompanies this BG opening (Table S5, Supporting Information). Although the computed BGs, expectedly underestimated by the semilocal approximations to the adopted XC‐functionals,^13^ are well beyond the visible light spectrum (1.6–3.1 eV), transfer of existing AlSi and AlGe Fe‐doping strategies^14^ to the considered AlSi‐Me NTs could be effective in reducing the AlSi‐Me BG to the visible range (2.2 eV in vacuo regardless of the NT diameter, owing to transitions between the VBE and empty Fe‐states, see Figures S6 and S7, Supporting Information). Furthermore, when gauged against demanding selectivity and separation requirements, use of UV light (>3.1 eV) may be profitably considered.^3^

Figure 2a compares the vacuum‐aligned AlSi_N_‐Me band edges with those of rutile and anatase TiO_2_, whose mixture is known to lead to effective water (H_2_O) photolysis.^15^ Notably, the AlSi_N_‐Me NTs VBEs are at least 1.59 eV (2.06 eV) higher than the rutile (anatase) VBE. Likewise, the AlSi_N_‐Me NTs CBEs are at least 3.07 eV (3.40 eV) higher than the rutile (anatase) CBE. The AlSi_N_‐Me NTs VBEs (CBEs) are also over 1 eV (4 eV) higher than those of TiO_2_(110) at the same level of theory (PBE[[qv: 15c]]). Neglecting the PC‐reactant‐medium interface structuring and electron transfer kinetics, these results suggest a lower (higher) H_2_O direct photo‐oxidation (reduction) drive for AlSi_N_‐Me NTs with respect to TiO_2_. While detrimental to their possible use as photo‐oxidant, the noticeably high‐energy VBEs of the AlSi_N_‐Me NTs (−6.24/−5.88 eV depending on the XC‐functional, Table S3, Supporting Information) suggest that grafting a molecular or nanoparticle PC to Imo‐NTs may be a rewarding strategy to enhance e‐h separation by promoting h‐transfer and relaxation from the grafted PC onto the NT. The upward energy shift of the AlSi_N_‐Me NTs VBE (CBE) with respect to the TiO_2_(110) results at the same level of theory (PBE, Figure 2a) suggest that our conclusions should be qualitatively unaffected by the expected limitations of the adopted XC‐functionals for absolute VBE (CBE) alignments.^16^

The weak dependence of the AlSi_N_‐Me BG and absolute band‐alignment on the NT‐diameter indicate that, while potentially beneficial for separation purposes, control of the NT‐diameter by varying the ionic strength of the synthetic solution^8^ does not allow effective band‐engineering for Imo‐NTs, at least for the considered NT‐composition and range of *N*.

Pristine AlSi and AlGe Imo‐NTs present an intriguing real‐space separation of the VBE and CBE,^11^ which may be beneficial for e–h separation via optical charge‐transfer excitations across the NT‐walls. We find this separation to be qualitatively unaffected by methylation, the diameter of the NTs, and adopted XC‐functional (Figure 2b and Figure S8, Supporting Information). Consistent with the real‐space distribution of the VBE and CBE, layer‐resolved analysis of the local density of states (LDOS in Figures S9–S14, Supporting Information) indicates major contributions of the C_2_/O_4_ (H_7_) layers to the VBE (CBE).

To explore the occurrence of optically active charge‐transfer excitations across the NT‐wall, we next simulate the optical spectra for the AlSi_N_‐Me systems (*N* = 28 and 34) bracketing the experimental pore‐size distribution.^6^ Notably, optical transitions involving states at the VBE and CBE on different sides of the NT‐cavity (Figure 2a) are found to contribute strongly to the low‐energy absorbance peak (Figure 2c), suggesting the occurrence of charge‐transfer excitations across the NT‐walls. These excitations and the prospective enhancement to e–h separation (the NT‐wall thickness is roughly 11 Å) may be beneficial to sustain photocatalytic reactivity. Such benefits should be larger for homogenous photocatalysis applications, with the soluble^6^ NTs dispersed in the same medium as the reactants, rather than deposited on a photo‐electrode. Provided no e (h) diffusion along the NT is needed by the photoreduction (oxidation) event, the NTs' relatively large effective electron (m_e_) and hole (m_h_) masses (m_e_: 0.79–0.81 m_0_, m_h_: 5.77–7.41 m_0_, Figures S19 and S20 and Table S9, Supporting Information) and expected low photoconductivity may not be a limitation. The experimentally observed photocatalytic reactivity for other porous aluminosilicate substrates^3^ of expected large electron (hole) mass supports this point. Given the limitations of the adopted XC‐functionals and approximations in evaluating the optical spectra, the transition energy is expected to be underestimated and possible excitonic effects missed.^17^ Nevertheless, the simulated optical activity for the (differently localized) states of the band‐edge should be meaningful.

Experimental and DFT results^11^ indicate that, due to accumulation of negative (positive) charge on the inner (outer) tube surface, the AlSi and AlGe walls are permanently polarized. The extent to which the wall‐polarization is jointly affected by the functionalization and curvature of the NT has not been previously considered for Imo‐NTs or, to the best of our knowledge, any other NT.

By application of Gauss' theorem to two coaxial hollow cylinders of (opposite) uniform charge density (see Supporting Information), the NT surface dipole density (*μ_σ_*) can be obtained from the difference $\left(\Delta \bar{V}={\bar{V}}_{\mathrm{in}}-{\bar{V}}_{\mathrm{out}}\right)$ between the plateaus of the angularly and longitudinally averaged (see Supporting Information, Equation (S10)) electrostatic (ionic plus Hartree) potential inside $\left({\bar{V}}_{\mathrm{in}}\right)$ and outside $\left({\bar{V}}_{\mathrm{out}}\right)$ the NT‐cavity

(1) $$\mu_{\sigma }=-\frac{\Delta \bar{V}}{4\pi }\frac{\Delta R}{R_{\mathrm{in}}}\frac{1}{\ln\left(\frac{R_{\mathrm{in}}}{R_{\mathrm{out}}}\right)}=-\frac{\Delta \bar{V}}{4\pi }\frac{\Delta R}{R_{\mathrm{in}}}\frac{1}{\ln\left(\frac{R_{\mathrm{in}}}{R_{\mathrm{in}}+\Delta R}\right)}$$

with the inner (*R*_in_) and outer (*R*_out_) radii defined as the onset of the vacuum‐electrostatic plateaus inside and outside the NT (**Figure** **3**a), that is, the radii where the vacuum oscillations of $\bar{V}$ are smaller than an arbitrary (5 × 10^–3^ eV) threshold. $\Delta R=R_{\mathrm{out}}-R_{\mathrm{in}}$ is the electrostatic thickness of the NT‐wall. For the sign convention used, positive *μ_σ_* values indicate accumulation of negative (positive) charge–density at the inner (outer) surface of the NTs, with creation of electronegative (electropositive) environments inside (outside) the NT‐cavity. As the NTs are enclosed by vacuum in the directions perpendicular to the NT‐axis, no filtering or nanosmoothing[[qv: 18a]] was applied. Given the overestimation of the electron‐density vacuum‐decay by GGA XC‐functionals,[[qv: 18b]] the computed *R*_in_ (*R*_out_) is to be taken as an upper (lower) bound to the exact value. Accordingly, for a constant (or of the same order of magnitude) XC‐error in *R*_in_ and *R*_out_ (*δR*), the analytical form of Equation (1) will return a lower bound to the correct *μ_σ_* for the same potential offset $\Delta \bar{V}$.

**Figure 3:**
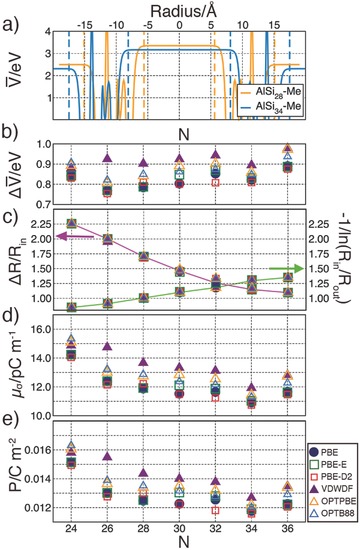
a) (PBE) average electrostatic potential ($\Delta \bar{V}$) along the NT radius for AlSi_28_‐Me and AlSi_34_‐Me. The dotted vertical lines mark the inner (*R*_in_) and outer (*R*_out_) NT radii as defined by the onset of the vacuum electrostatic plateaus. b) Computed NT‐wall potential step $\Delta \bar{V}$, c) geometric factors $\frac{\Delta R}{R_{\mathrm{in}}}$ and ${\left[-\ln\left(\frac{R_{\mathrm{in}}}{R_{\mathrm{out}}}\right)\right]}^{-1}$, d) surface dipole density *μ_σ_*, and e) polarization *P* for the AlSi_N_‐Me NTs as a function of *N* and the XC‐functional.

The potential step, geometric factors and *μ_σ_* for AlSi_N_‐Me are shown in Figure 3b–d. We find a small dependence (<0.15 eV) of $\Delta \bar{V}$ on both *N* (NT radius) and the functional used, with explicitly nonlocal dispersion XC‐functionals (VDWDF, OPTPBE, and OPTB88) yielding slightly larger potential steps than (empirically corrected) PBE (Figure 3b). The sub‐meV convergence of the change of $\Delta \bar{V}$ with N as a function of the psinc‐grid energy cutoff and NGWFs‐radius rules out any numerical artefact in the computed trends (Figures S15 and S16 and Table S7, Supporting Information). Therefore, despite the roughly constant $\Delta \bar{V}$, and due to the decrease of the $\frac{\Delta R}{R_{\mathrm{in}}}$ geometric factor with the NT‐radius (*N*) dominating the increase of the ${\left[-\ln\left(\frac{R_{\mathrm{in}}}{R_{\mathrm{out}}}\right)\right]}^{-1}$ term (Figure 3c), *μ_σ_* is found to decrease for AlSi_N_‐Me NTs of larger radius (Figure 3d). Thus, the walls of the larger NTs are less polarized than the smaller ones. The positive value of *μ_σ_* indicates that, as for the pristine AlSi and AlGe systems,^11^ the AlSi_N_‐Me NTs present accumulation of negative (positive) inside (outside) the NT‐cavity. The decrease of *μ_σ_* with *N* is coupled with a depletion of negative charge on the inner methyl group as *N* increases (Table S5, Supporting Information).

In line with chemical intuition, substitution of stronger electron‐withdrawing hydroxyls by methyl groups inside AlSi_24_ reduces *μ_σ_* substantially (−42%) from 22.48 pC m^–1^ (AlSi_24_) to 14.24 pC m^−1^ (AlSi_24_‐Me, Table S6, Supporting Information). Based on the observed correspondence between system polarization and favorable e–h separation,[[qv: 5b–d]] the latter should be enhanced in pristine (not methylated) AlSi NTs. Maximization of *μ_σ_* toward enhanced e–h separation in Imo‐NTs should accordingly target use of strongly electron‐attracting (donating) substituents on the inner (outer) side the NT cavity. To this end, use of chloromethyl‐ or other halogenated substituents, rather than methyl‐silane precursors,^6^ may be profitably explored to enhance *μ_σ_* while maintaining the overall hydrophobic cavity in hybrid organic–inorganic Imo‐NTs. In this respect, trifluoromethylated AlSi_N_‐CF_3_ NTs are computed to have a fourfold increased *μ_σ_* (44.52–56.50 pC m^–1^, depending on *N* and the XC‐functional, Table S8, Supporting Information), with preserved VBE–CBE separation (Figure S17, Supporting Information), and a 0.4–0.6 eV reduction of the BG owing to an upward shift of the VBE (Table S8, Supporting Information).

To quantitatively discuss the charge‐separation in AlSi‐Me NTs with respect to state of the art photo‐ferroelectrics, and provide a better estimate their e–h separation propensity, we next turn to the charge‐separation per unit of (medium‐excluded) volume, i.e., the polarization, *P*. To this end, we integrate the surface dipole density (*μ_σ_*) over the surface of the NT dipole‐layer (*S* = *2πR_av_L*, *L* is the tube length) calculated from the average electrostatic radius [*R_av_* = *½*(*R*_in_ + *R*_out_), taken as the center of the warped dipole layer]. Dividing the result by the tube volume [*V* = π(*R*_out_^2^ *–R*_in_^2^)*L*] yields the NT‐dipole per unit of volume, i.e., the polarization, *P*:

(2) P=μσSV=μσ2π12(R+inRout)Lπ(Rout2−Rin2)L=μσ(R+inR+inΔR)[(R+inΔR)2−Rin2]=μσΔR=−ΔV¯4π1Rin1ln(RinRin+ΔR)

Figure 3e shows that the AlSi_N_‐Me polarization is one order of magnitude smaller than for standard photo‐ferroelectrics (BaTiO_3_: 0.26–0.34 C m^–2^,[[qv: 19a]] KNbO_3_: 0.55 C m^–2^[[qv: 19b]]). Given the roughly constant Δ*R* for the considered AlSi_N_‐Me (Table S6, Supporting Information), the dependence of *P* and *μ_σ_* on *N* (NT‐radius) is very similar.

Notably, the AlSi_N_‐Me polarization is achieved by way of abundant and light elements (H, C, O, Al, Si) in a warped layer roughly 1 nm thick whereby state of the art cubic photocatalytic BaTiO_3_ nanoparticles have sides on the order of 7.5 nm.[[qv: 5c]] This may be beneficial for cost‐effective use of materials in creating polarized interfaces. In addition, the non‐negligible *P*‐values for AlSi_N_‐Me NTs suggest use of overall dipole‐free 1D nanostructures with chemically heterogeneous ≈1 nm thick walls as an effective strategy to circumvent critical thickness issues in ferroelectric substrates.^20^

Besides being potentially beneficial for e–h separation, the NTs *μ_σ_* leads to markedly different electrostatic environments on either side of the NT‐cavity ($\Delta \bar{V}\neq 0$). This can used to modulate NT‐reactants (or nanoconfined photocatalyst‐reactant) electronic alignments and affect e (h) transfer kinetics.^1^, ^2^ Equation (1) compactly provides directions for future synthetic efforts aimed at increasing *μ_σ_* (to the benefit of e–h separation) while simultaneously influencing the NT‐reactant electronic alignment toward enhanced e (h) transfer kinetics. Owing to the geometric factors in Equation (1), the same surface dipole density (*μ_σ_*) differently arranged in space can lead to a different potential step across the NT‐wall ($\Delta \bar{V}$). As shown in Figure S4 of the Supporting Information, large *R*_in_ and small Δ*R* values allow maximization of the potential difference ($\Delta \bar{V}$) for a given surface dipole density (μ_σ_). Conversely, the same μ_σ_ can lead to smaller $\Delta \bar{V}$ values, provided *R*_in_ (Δ*R*) is decreased (increased). The extent to which this result is affected by the presence of a medium of variable ionic strength remains to be quantified and requires further research, which we hope to stimulate with this work.

In conclusion, linear‐scaling DFT with six different semilocal and dispersion‐corrected functionals has been used to elucidate the interplay between chemical functionalization, curvature, local permanent polarizations, band gap, band‐separation, band‐alignment, and the occurrence of charge‐transfer excitations in an existing class of hybrid organic–inorganic nanotubes with hydrophobic interior and hydrophilic exterior: methylated aluminosilicate imogolite NTs. Strategies based on the generated insight have been suggested to increase the NT polarization to values comparable with state of the art ferroelectric photocatalysts, and to tune NT‐reactant electronic alignments by altering the NT radius and wall‐thickness. We hope these results on the potential of (hybrid organic–inorganic) nanotubes for polarization‐enhanced photocatalytic applications will stimulate further experimental interest and investigations.

## Experimental Section

All the simulations were performed with the ONETEP program.^21^ Following benchmarks,^22^ four (one) 8 bohrs valence and conduction nonorthogonal generalized Wannier functions (NGWFs^21^) were used for Al, Si, C, O (H) atoms. The psinc basis set energy cutoff^21^ was 1 000 eV. No truncation of the density kernel was enforced. Separable (Kleinman–Bylander) norm‐conserving pseudopotentials^23^ and periodic boundary conditions, with 15 Å of vacuum padding between replicated images in the nonperiodic directions, were used. The convergence thresholds for NGWFs and geometry optimization were 10^–4^ eV per atom and 0.05 eV Å^−1^, respectively. The optimized length of the NT‐repeat unit (8.666 Å) was found to be constant for the explored range of *N* and XC‐functionals used (Figure S21, Supporting Information). Conduction NGWFs were optimized following Ratcliff et al.^24^ Given the known deficiencies of time‐dependent DFT in the adiabatic local‐density approximation (ALDA) in the description of periodic systems as considered here,^17^ optical spectra were approximated via the Fermi Golden rule approach described by Ratcliff et al.^24^ To investigate possible deficiencies of the PBE functional[[qv: 25a]] due to the presence of polarizable methyl groups, different treatments of dispersion interactions were considered: Grimme (PBE‐D2)[[qv: 25b]] and Elstner (PBE‐E)[[qv: 25c–d]] empirical corrections as well as three self‐consistent dispersion functionals: VDWDF,[[qv: 25e–f]] OPTPBE,[[qv: 25g]] and OPTB88,[[qv: 25g]] which differ in the treatment of the exchange contribution only.

## Supporting information

As a service to our authors and readers, this journal provides supporting information supplied by the authors. Such materials are peer reviewed and may be re‐organized for online delivery, but are not copy‐edited or typeset. Technical support issues arising from supporting information (other than missing files) should be addressed to the authors.

Supplementary

## References

[1] a) P. V. Kamat , J. Phys. Chem. C 2007, 111, 2834;

[2] a) S. N. Habisreutinger , L. Schmidt‐Mende , J. K. Stolarczyk , Angew. Chem. Int. Ed. 2013, 52, 7372; 10.1002/anie.201207199 23765842

[3] a) F. Sastre , V. Fornes , A. Corma , H. García , J. Am. Chem. Soc. 2011, 133, 17257; 21939273 10.1021/ja204559z

[4] a) H. Tong , S. Ouyang , Y. Bi , N. Umezawa , M. Oshikiri , J. H. Ye , Adv. Mater. 2012, 24, 229; 21972044 10.1002/adma.201102752

[5] a) J. Kreisel , M. Alexe , P. A. Thomas , Nat. Mater. 2012, 11, 260; 22437772 10.1038/nmat3282

[6] a) I. Bottero , B. Bonelli , S. E. Ashbrook , P. A. Wright , W. Zhou , M. Tagliabue , M. Armandi , E. Garrone , Phys. Chem. Chem. Phys. 2011, 13, 744; 21046043 10.1039/c0cp00438c

[7] P. D. Cradwick , K. Wada , J. D. Russell , N. Yoshinaga , C. R. Masson , V. C. Farmer , Nat. Phys. Sci. 1972, 240, 187.

[8] a) S. I. Wada , A. Eto , K. Wada , J. Soil Sci. 1979, 30, 347;

[9] a) P. Maillet , C. Levard , E. Larquet , C. Mariet , O. Spalla , N. Menguy , A. Masion , E. Doelsch , J. Rose , A. Thill , J. Am. Chem. Soc. 2010, 132, 1208; 20055384 10.1021/ja908707a

[10] a) D. Y. Kang , J. Zang , C. W. Jones , S. Nair , J. Phys. Chem. C 2011, 115, 7676;

[11] a) J. P. Gustafsson , Clays Clay Miner. 2001, 49, 73;

[12] a) A. J. Bard , J. Photochem. 1979, 10, 59;

[13] a) J. P. Perdew , R. G. Parr , M. Levy , J. L. Balduz , Phys. Rev. Lett. 1982, 49, 1691;

[14] a) M. Ookawa , in Clay Minerals in Nature – Their Characterization, Modification and Application (Ed: Valaskova M.), InTech, Rijeka, Croatia, 2012, p. 2708.

[15] a) D. O. Scanlon , C. W. Dunnill , J. Buckeridge , S. A. Shevlin , A. J. Logsdail , S. M. Woodley , C. R. A. Catlow , M. J. Powell , R. G. Palgrave , I. P. Parkin , G. W. Watson , T. W. Keal , P. Sherwood , A. Walsh , A. A. Sokol , Nat. Mater. 2013, 12, 798; 23832124 10.1038/nmat3697

[16] a) J. P. Perdew , R. G. Parr , M. Levy , J. L. Balduz , Phys. Rev. Lett. 1982, 49, 1691;

[17] G. Onida , L. Reining , A. Rubio , Rev. Mod. Phys. 2002, 74, 601. 10.1103/PhysRevLett.88.066404 11863831

[18] a) J. Junquera , M. H. Cohen , K. M. Rabe , J. Phys.: Condens. Matter 2007, 19, 213204;

[19] a) J. J. Wang , F. Y. Meng , X. Q. Ma , M. X. Xu , L. Q. Chen , J. Appl. Phys. 2010, 108, 034107;

[20] a) J. Junquera , P. Ghosez , Nature 2003, 422, 506; 12673246 10.1038/nature01501

[21] a) C.‐K. Skylaris , P. D. Haynes , A. A. Mostofi , M. C. Payne , J. Chem. Phys. 2005, 122, 084119; 10.1063/1.1839852 15836032

[22] E. Poli , J. D. Elliott , N. D. M. Hine , A. A. Mostofi , G. Teobaldi , Mater. Res. Innov. 2015, 19, S272.

[23] X. Gonze , R. Stumpf , M. Scheffler , Phys. Rev. B 1991, 44, 8503. 10.1103/physrevb.44.8503 9998805

[24] L. E. Ratcliff , N. D. M. Hine , P. D. Haynes , Phys. Rev. B 2011, 84, 165131.

[25] a) J. Perdew , K. Burke , M. Ernzerhof , Phys. Rev. Lett. 1996, 77, 3865; 10062328 10.1103/PhysRevLett.77.3865

