# Supplementary material for: Chemically Selective Alternatives to Photoferroelectrics for Polarization‐Enhanced Photocatalysis: The Untapped Potential of Hybrid Inorganic Nanotubes
Source: Adv Sci (Weinh). 2016 Sep 13;4(2):1600153. doi: 10.1002/advs.201600153 (PMC5323885; doi:10.1002/advs.201600153)
Supplement: Supplementary file 1 — Supplementary [file ADVS-4-na-s001.pdf]

Copyright WILEY-VCH Verlag GmbH & Co. KGaA, 69469 Weinheim, Germany, 2016.

ADVANCED SCIENCE
Open Access

Supporting Information

for *Adv. Sci.,* DOI: 10.1002/advs.201600153

Chemically Selective Alternatives to Photoferroelectrics for Polarization-Enhanced Photocatalysis: The Untapped Potential of Hybrid Inorganic Nanotubes

*Joshua D. Elliott, Emiliano Poli, Ivan Scivetti, Laura E. Ratcliff, Lampros Andrinopoulos, Jacek Dziedzic, Nicholas D. M. Hine, Arash A. Mostofi, Chris-Kriton Skylaris, Peter D. Haynes, and Gilberto Teobaldi**

Copyright WILEY-VCH Verlag GmbH & Co. KGaA, 69469 Weinheim, Germany, 2013.

Supporting Information

Chemically Selective Alternatives to Photoferroelectrics for Polarization-Enhanced Photocatalysis: the Untapped Potential of Hybrid Inorganic Nanotubes

*Joshua D. Elliott, Emiliano Poli, Ivan Scivetti, Laura E. Ratcliff, Lampros Andrinopoulos, Jacek Dziedzic, Nicholas D. M. Hine, Arash A. Mostofi, Chris-Kriton Skylaris, Peter D. Haynes, Gilberto Teobaldi**

1. Supplementary methods

Potential step in a co-axial cylindrical capacitor

As shown in Figure 3a, in spite of the NT-wall polarization, and owing to the NT cylindrical symmetry and overall charge-neutrality, the NTs present a flat electrostatic potential [$V(\mathbf{r})$] inside and outside the NT-cavity. Since the electrostatic field ($\vec{\mathbf{E}}$) is given by the negative gradient of the electrostatic potential [$\vec{\mathbf{E}} = -\nabla V(\mathbf{r})$], no electrostatic field is present inside and outside the NT. As a result, it is possible to model the NT electrostatics on the basis of an overall neutral co-axial (hollow) cylindrical capacitor (Figure S1).

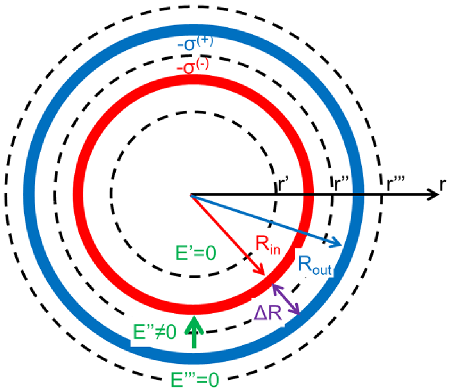

Figure S1. Front view of a cylindrical co-axial capacitor with charge $-Q = -\sigma^{(-)} 2\pi R_{in} L$ and $Q = \sigma^{(+)} 2\pi R_{out} L$ on the inner and outer hollow cylinder, respectively. L is the length of the inner and outer hollow cylinders. The three cylindrical Gaussian surfaces of radius r', r'', and r''' are also indicated.

Gauss' theorem relates the flux of the electrostatic field ($\vec{\mathbf{E}}$) across a closed surface (S) to the charge Q contained inside the closed surface:

$$\frac{Q}{\varepsilon_0} = \oint_S \vec{\mathbf{E}} \cdot \mathbf{d\hat{s}} \tag{S1}$$

where ε_0 is the electric permittivity of vacuum, and $\mathbf{d\hat{s}}$ a vector of unitary module locally normal to the infinitesimal surface element. Eq. S1 allows definition of three electrostatic regions with different in the co-axial capacitor in Figure S1:

Region 1. For $r < R_{in}$, the electric field is zero ($E = 0$) since the Gaussian surface of radius r' does not contain any net charge ($Q = 0$).

Region 2. For $R_{in} \le r < R_{out}$, the electric field is not zero ($E \neq 0$) since the Gaussian surface of radius r'' does contain a net charge ($-Q < 0$, see Figure S1). As the hollow cylinders are taken to be in electrostatic equilibrium, with no net transfer of charge, $\mathbf{E} = E\hat{\mathbf{r}}$ i.e. the electrostatic field must lie parallel to the tube radius, with zero components along the tube (lateral) surface.

Region 3. For $r > R_{out}$, the electric field is zero ($E = 0$) since the Gaussian surface of radius r''' does not contain any net charge ($Q = 0$), being the cylindrical capacitor overall neutral.

We thus focus in Region 2 to calculate the electric field and potential difference between R_{in} and R_{out} by Gauss' flux theorem. We start by expanding both sides of Eq. S1 as:

$$\frac{Q}{\varepsilon_0} = \frac{-2\pi R_{in} L \sigma^{(-)}}{\varepsilon_0} = \oint_S \vec{\mathbf{E}} \cdot \mathbf{d\hat{s}} = 2\pi r'' L E \tag{S2}$$

Where in the right-side term we have taken advantage of $\vec{\mathbf{E}}$ being locally parallel to $\mathbf{d\hat{s}}$ and that the $\vec{\mathbf{E}}$ between the cylinders has to be directed parallel to the surface normal with zero components along the cylinder axis.

Eq. S2 can be rearranged to read:

$$\frac{-R_{in}\sigma^{(-)}}{\varepsilon_0 r''} = E = -\frac{dV}{dr''} \tag{S3}$$

which allows the integration of the electrostatic potential between R_{in} and R_{out} as:

$$\Delta V = V(R_{in}) - V(R_{out}) = \int_{R_{out}}^{R_{in}} dr'' E = \frac{R_{in}\sigma^{(-)}}{\varepsilon_0} \int_{R_{out}}^{R_{in}} dr'' \frac{1}{r''} = \frac{R_{in}\sigma^{(-)}}{\varepsilon_0} \ln\left(\frac{R_{in}}{R_{out}}\right) \tag{S4}$$

It is worth noting that, in Eq. S4, the overall negative sign of $\Delta V = V(R_{in}) - V(R_{out})$ [$\ln\left(R_{in}/R_{out}\right) < 0$] is consistent with $\vec{\mathbf{E}}$ being directed from the outer (positively charged) to the inner (negatively charged) cylinder (Figure S1).

In analogy with the treatment for the surface dipole density (μ_σ) due to two charged surfaces (of surface charge-density σ) locally parallel and separated by a distance *d*:[S1]

$$\lim_{d \to 0} \sigma d = \mu_\sigma \tag{S5}$$

For infinitesimally small separation ΔR, leading to $R_{in} = R_{out}$ and $\sigma^{(-)} = \sigma^{(+)} = \sigma$, the separation in surface charge density (σ) between the inner and outer cylinders (Figure S2) can be described via a surface dipole-density as:

$$\lim_{\Delta R \to 0} \sigma \Delta R = \mu_\sigma \tag{S6}$$

which in turn can be used to write:

$$\sigma = \frac{\mu_\sigma}{\Delta R} \tag{S7}$$

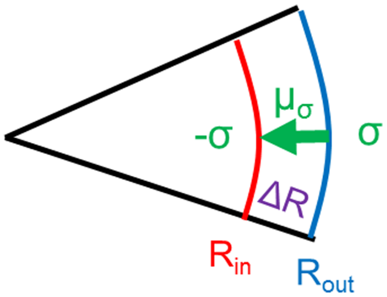

Figure S2. Separation of charge density (σ) between the co-axial cylinders, leading to a surface dipole-density μ_σ.

Although Eq. S5 is strictly verified for infinitesimally small separations between the charge-layers (*d*), it is routinely used in the modelling of potential steps across atomically heterogeneous bi-dimensional junctions (of finite thickness) between different materials (see, for instance, Ref. [S2] and references therein). Therefore, by using Eq. S6, we resort to the same approximation in computing the potential step across the interface dipole at the NT-wall of atomically finite thickness $\Delta R \neq 0$. Accordingly, and based on Eq. S7, Eq. S4 can be rearranged into:

$$\Delta V = V(R_{in}) - V(R_{out}) = \frac{R_{in}\sigma}{\varepsilon_0} \ln\left(\frac{R_{in}}{R_{out}}\right) = \frac{R_{in}}{\varepsilon_0} \frac{\mu_\sigma}{\Delta R} \ln\left(\frac{R_{in}}{R_{out}}\right) = 4\pi R_{in} \frac{\mu_\sigma}{\Delta R} \ln\left(\frac{R_{in}}{R_{in} + \Delta R}\right) \tag{S8}$$

where we have used the fact that in atomic units $1/\varepsilon_0 = 4\pi$.

For consistency with the convention in some DFT-codes of calculating the electrostatic potential (Figure 3a) using the (negatively charged) electron as test charge, leading to lower (higher) electrostatic potential for electron-rich (poor) regions, the sign of Eq. S8 needs to be changed leading to:

$$\Delta V = V(R_{in}) - V(R_{out}) = -4\pi R_{in}\frac{\mu_\sigma}{\Delta R}\ln\left(\frac{R_{in}}{R_{out}}\right) = -4\pi R_{in}\frac{\mu_\sigma}{\Delta R}\ln\left(\frac{R_{in}}{R_{in}+\Delta R}\right) \quad \text{(S9)}$$

This correctly describes regions of high (low) electrostatic potential for the electron-rich (poor) side of the NT-cavity (Figure 3a). Eq. S8 allows computation of dipole-density from the step in the electrostatic potential across the NT-wall. Given the solution to the DFT problem via discretized grids,[S3] the non-homogeneous electrostatic potential inside (and immediately outside) any material, and in analogy with standard procedure for planar dipole densities,[S2] it is convenient to angularly and longitudinally average the electrostatic potential (expressed in cylindrical coordinates):

$$\bar{V}(r) = \frac{1}{2\pi L}\int_0^{2\pi} d\phi \int_0^{L} dl\, V(r,\phi,l) \quad \text{(S10)}$$

obtaining:

$$\Delta\bar{V} = \bar{V}(R_{in}) - \bar{V}(R_{out}) = -4\pi R_{in}\frac{\mu_\sigma}{\Delta R}\ln\left(\frac{R_{in}}{R_{out}}\right) = -4\pi R_{in}\frac{\mu_\sigma}{\Delta R}\ln\left(\frac{R_{in}}{R_{in}+\Delta R}\right) \quad \text{(S11)}$$

This last equation is used to compute μ_σ on the basis of the potential step ($\Delta\bar{V}$) between the electrostatically derived R_{in} and R_{out} (Figure 3).

It is worth noting that, for increasingly large R_{in} ($R_{out} = R_{in} + \Delta R$), the cylindrical capacitor asymptotically tends to a planar one, and Eq. S11 asymptotically recovers the established $\frac{\Delta\bar{V}}{\mu_\sigma} = 4\pi$ relationship (Figure S3) for the potential step due to a planar dipole density.[S1]

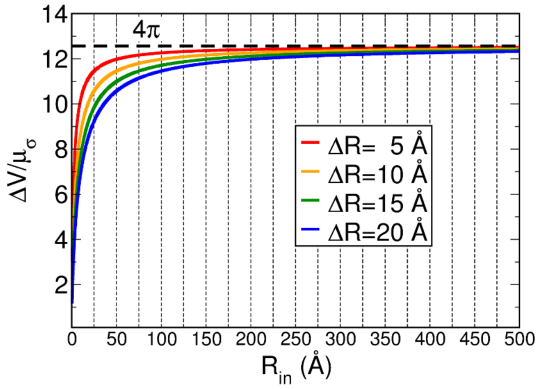

Figure S3. Asymptotic behavior of $\frac{\Delta\bar{V}}{\mu_\sigma} = -4\pi\frac{R_{in}}{\Delta R}\ln\left(\frac{R_{in}}{R_{in}+\Delta R}\right)$ (obtained from Eq. S11) for increasing values of R_{in} and ΔR.

Eq. S10 allows exploring the role of the geometric factors and the interplay between R_{in} and ΔR in damping the relationship between surface dipole-density μ_σ and potential step across the NT-wall $\Delta\bar{V}$. As shown in Figure S4, Large R_{in} and small ΔR values allow

maximization of the potential difference ($\Delta\overline{V}$) for a given surface dipole-density (μ_σ). Conversely, smaller $\Delta\overline{V}$ values can be obtained for the same μ_σ provided R_{in} (ΔR) is decreased (increased).

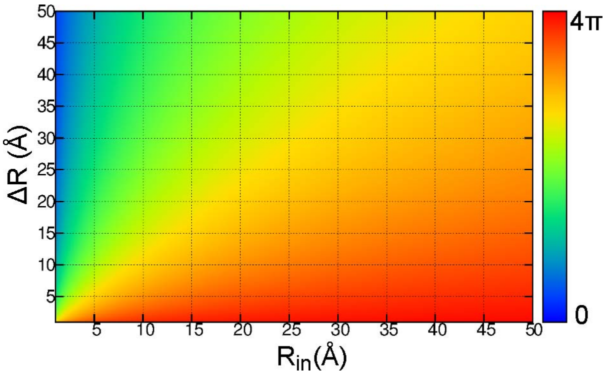

Figure S4. Two-dimensional plot of $\frac{\Delta\overline{V}}{\mu_\sigma} = -4\pi \frac{R_{in}}{\Delta R} \ln\left(\frac{R_{in}}{R_{in}+\Delta R}\right)$ (obtained from Eq. S11) as a function of R_{in} and ΔR.

Band structure calculations

Band structure calculations were performed via the Projected Augmented Wave (PAW) method as implemented in the VASP program [S4], with the PBE XC-functional [S5], a 400 eV plane wave energy cutoff, 0.1 eV Gaussian smearing, and 10 k-points along the reciprocal periodic direction of the NTs.

As common practice [S6], effective electron (hole) mass were computed via parabolic fitting at the bottom (top) of the computed conduction (valence) band, with wavevector (**k**) fitting ranges small enough to ensure fitting errors of less than 0.5%.

2. Supplementary results

Table S1. Average atom-resolved diameters and standard deviation (Å) for the optimized NT-models and considered XC-functionals. The adopted labeling corresponds to the atom element and the subscript-suffix numbers the radial layer (see also Figure 1a). *N* is the number of radially non-equivalent Al-atoms contained within the NT circumference.

	H_1 [Å]	C_2 [Å]	Si_3 [Å]	O_4 [Å]	Al_5 [Å]	O_6 [Å]	H_7 [Å]
**N = 24**							
PBE	12.48±0.01	13.24±0.01	16.90±0.01	18.17±0.04	20.08±0.01	22.25±0.05	23.45±0.04
PBE-E	12.48±0.01	13.24±0.01	16.90±0.01	18.17±0.04	20.08±0.01	22.25±0.05	23.45±0.04
PBE-D2	12.48±0.01	13.24±0.01	16.90±0.01	18.17±0.04	20.08±0.01	22.25±0.05	23.45±0.04
VDWDF	12.48±0.01	13.24±0.01	16.90±0.01	18.17±0.04	20.08±0.01	22.24±0.05	23.46±0.04
OPTPBE	12.48±0.01	13.24±0.01	16.90±0.01	18.17±0.04	20.08±0.01	22.24±0.05	23.45±0.04
OPTB88	12.48±0.01	13.24±0.01	16.90±0.01	18.17±0.04	20.08±0.01	22.24±0.05	23.45±0.04
**N = 26**							
PBE	13.73±0.00	14.48±0.00	18.15±0.00	19.43±0.04	21.36±0.00	23.54±0.04	24.71±0.05
PBE-E	13.73±0.00	14.48±0.00	18.15±0.00	19.43±0.04	21.36±0.00	23.54±0.04	24.71±0.05
PBE-D2	13.73±0.00	14.49±0.00	18.15±0.00	19.43±0.04	21.36±0.00	23.53±0.04	24.71±0.05
VDWDF	13.73±0.00	14.49±0.00	18.15±0.00	19.43±0.04	21.37±0.00	23.53±0.04	24.72±0.05
OPTPBE	13.73±0.00	14.50±0.00	18.16±0.00	19.44±0.04	21.36±0.00	23.53±0.04	24.72±0.05
OPTB88	13.73±0.00	14.50±0.00	18.16±0.00	19.43±0.04	21.36±0.00	23.52±0.04	24.71±0.04
**N = 28**							
PBE	15.33±0.03	16.10±0.02	19.77±0.01	21.04±0.04	22.97±0.01	25.15±0.05	26.33±0.03
PBE-E	15.33±0.03	16.10±0.02	19.77±0.01	21.04±0.04	22.97±0.01	25.15±0.05	26.33±0.03
PBE-D2	15.35±0.01	16.11±0.01	19.78±0.00	21.04±0.04	22.97±0.01	25.14±0.04	26.32±0.02
VDWDF	15.34±0.01	16.11±0.01	19.78±0.01	21.05±0.03	22.98±0.01	25.14±0.04	26.35±0.03
OPTPBE	15.34±0.02	16.11±0.01	19.77±0.01	21.04±0.03	22.97±0.01	25.14±0.04	26.33±0.03
OPTB88	15.34±0.02	16.11±0.01	19.78±0.01	21.04±0.03	22.97±0.01	25.13±0.04	26.33±0.03
**N = 30**							
PBE	16.93±0.02	17.68±0.01	21.35±0.00	22.61±0.05	24.55±0.00	26.72±0.04	27.90±0.05
PBE-E	16.93±0.01	17.70±0.01	21.39±0.01	22.64±0.03	24.58±0.01	26.76±0.04	27.95±0.02
PBE-D2	16.94±0.01	17.69±0.01	21.36±0.00	22.61±0.04	24.54±0.00	26.71±0.04	27.88±0.04
VDWDF	16.97±0.02	17.74±0.02	21.42±0.02	22.67±0.04	24.61±0.02	26.77±0.04	27.98±0.03
OPTPBE	16.96±0.02	17.73±0.02	21.41±0.02	22.65±0.04	24.58±0.02	26.75±0.04	27.94±0.03
OPTB88	16.93±0.01	17.69±0.01	21.35±0.00	22.61±0.05	24.53±0.00	26.70±0.04	27.89±0.05
**N = 32**							
PBE	18.55±0.02	19.32±0.02	23.01±0.01	24.26±0.03	26.19±0.01	28.37±0.04	29.56±0.03
PBE-E	18.53±0.02	19.30±0.02	22.99±0.01	24.24±0.03	26.18±0.01	28.36±0.04	29.55±0.01
PBE-D2	18.53±0.03	19.29±0.02	22.96±0.00	24.20±0.05	26.14±0.01	28.32±0.04	29.49±0.06
VDWDF	18.55±0.02	19.32±0.02	23.01±0.02	24.26±0.04	26.20±0.02	28.36±0.04	29.56±0.02
OPTPBE	18.56±0.02	19.32±0.02	23.00±0.01	24.24±0.04	26.16±0.02	28.33±0.04	29.52±0.02
OPTB88	18.54±0.05	19.30±0.02	22.96±0.00	24.21±0.05	26.13±0.01	28.30±0.03	29.50±0.05
**N = 34**							
PBE	20.11±0.01	20.88±0.01	24.56±0.01	25.79±0.04	27.73±0.01	29.91±0.03	31.07±0.04
PBE-E	20.12±0.01	20.88±0.01	24.56±0.01	25.79±0.04	27.73±0.01	29.90±0.03	31.07±0.04
PBE-D2	20.11±0.01	20.87±0.01	24.56±0.01	25.80±0.03	27.73±0.01	29.90±0.03	31.07±0.04
VDWDF	20.10±0.01	20.87±0.01	24.56±0.01	25.80±0.03	27.74±0.01	29.91±0.03	31.10±0.04
OPTPBE	20.12±0.01	20.88±0.01	24.56±0.01	25.80±0.04	27.73±0.01	29.90±0.03	31.08±0.04
OPTB88	20.12±0.01	20.89±0.01	24.56±0.01	25.80±0.04	27.72±0.01	29.89±0.03	31.07±0.04
**N = 36**							
PBE	21.69±0.02	22.46±0.01	26.15±0.01	27.38±0.04	29.32±0.01	31.50±0.04	32.70±0.05
PBE-E	21.69±0.02	22.46±0.01	26.15±0.01	27.38±0.04	29.32±0.01	31.50±0.04	32.70±0.05
PBE-D2	21.72±0.00	22.48±0.01	26.16±0.01	27.38±0.04	29.32±0.01	31.49±0.03	32.67±0.03
VDWDF	21.70±0.01	22.47±0.01	26.15±0.01	27.39±0.03	29.33±0.01	31.50±0.03	32.71±0.04
OPTPBE	21.71±0.01	22.48±0.01	26.16±0.01	27.39±0.04	29.32±0.01	31.49±0.03	32.70±0.04
OPTB88	21.70±0.01	22.47±0.01	26.15±0.01	27.39±0.04	29.32±0.01	31.48±0.03	32.70±0.05

Table S2. Average Layer-resolved bond lengths and their standard deviations (Å) for optimized NT-models and considered XC-functionals. The adopted labeling corresponds to the atom element and the subscript-suffix numbers the radial layer (see also Figure 1a). *N* is the number of radially non-equivalent Al-atoms in the NT circumference. The PBE results for the pristine $AlSi_{24}$ NT are reported for comparison.

	H_1-$C(O)_2$ [Å]	$C(O)_2$-$Si(Ge)_3$ [Å]	$Si(Ge)_3$-O_4 [Å]	O_4-Al_5 [Å]	Al_5-O_6 [Å]	O_6-H_7 [Å]
**$AlSi_{24}$ (PBE)**	0.97±0.00	1.65±0.00	1.65±0.01	1.94±0.02	1.89±0.01	0 .96±0.00
**N = 24**						
PBE	1.09±0.00	1.84±0.00	1.66±0.01	1.95±0.01	1.90±0. 01	0.96±0.00
PBE-E	1.09±0.00	1.84±0.00	1.66±0.01	1.95±0.01	1.90± 0.01	0.96±0.00
PBE-D2	1.09±0.00	1.84±0.00	1.66±0.01	1.95±0.01	1.90 ±0.01	0.96±0.00
VDWDF	1.08±0.00	1.84±0.00	1.66±0.01	1.95±0.01	1.90± 0.01	0.96±0.00
OPTPBE	1.09±0.00	1.84±0.00	1.66±0.01	1.95±0.01	1.90 ±0.01	0.96±0.00
OPTB88	1.09±0.00	1.84±0.00	1.66±0.01	1.95±0.01	1.90 ±0.01	0.96±0.00
**N = 26**						
PBE	1.09±0.00	1.85±0.00	1.66±0.01	1.94±0.01	1.89±0. 01	0.96±0.00
PBE-E	1.09±0.00	1.85±0.00	1.66±0.01	1.94±0.01	1.89± 0.01	0.96±0.00
PBE-D2	1.09±0.00	1.85±0.00	1.66±0.01	1.94±0.01	1.89 ±0.01	0.96±0.00
VDWDF	1.08±0.00	1.83±0.00	1.66±0.01	1.95±0.01	1.89± 0.01	0.96±0.00
OPTPBE	1.09±0.00	1.85±0.00	1.66±0.01	1.94±0.01	1.89 ±0.01	0.96±0.00
OPTB88	1.09±0.00	1.84±0.00	1.66±0.01	1.94±0.01	1.89 ±0.01	0.96±0.00
**N = 28**						
PBE	1.09±0.00	1.85±0.00	1.66±0.01	1.94±0.01	1.89±0. 01	0.96±0.00
PBE-E	1.09±0.00	1.85±0.00	1.66±0.01	1.94±0.01	1.89± 0.01	0.96±0.00
PBE-D2	1.09±0.00	1.85±0.00	1.66±0.01	1.94±0.01	1.89 ±0.01	0.96±0.00
VDWDF	1.09±0.00	1.85±0.00	1.66±0.01	1.95±0.01	1.89± 0.01	0.96±0.00
OPTPBE	1.09±0.00	1.85±0.00	1.66±0.01	1.94±0.01	1.89 ±0.01	0.96±0.00
OPTB88	1.09±0.00	1.85±0.00	1.66±0.01	1.94±0.01	1.89 ±0.01	0.96±0.00
**N = 30**						
PBE	1.09±0.00	1.85±0.00	1.66±0.01	1.95±0.01	1.89±0. 01	0.96±0.00
PBE-E	1.09±0.00	1.85±0.00	1.66±0.01	1.95±0.01	1.89± 0.01	0.96±0.00
PBE-D2	1.09±0.00	1.85±0.00	1.66±0.01	1.94±0.01	1.89 ±0.01	0.96±0.00
VDWDF	1.09±0.00	1.85±0.00	1.66±0.01	1.95±0.01	1.89± 0.01	0.96±0.00
OPTPBE	1.09±0.00	1.85±0.00	1.66±0.01	1.95±0.01	1.89 ±0.01	0.96±0.00
OPTB88	1.09±0.00	1.85±0.00	1.66±0.01	1.94±0.01	1.89 ±0.01	0.96±0.00
**N = 32**						
PBE	1.09±0.00	1.85±0.00	1.67±0.00	1.95±0.01	1.89±0. 01	0.96±0.00
PBE-E	1.09±0.00	1.85±0.00	1.67±0.01	1.95±0.01	1.89± 0.01	0.96±0.00
PBE-D2	1.09±0.00	1.85±0.00	1.67±0.01	1.95±0.01	1.89 ±0.01	0.96±0.00
VDWDF	1.09±0.00	1.85±0.00	1.66±0.01	1.95±0.01	1.89± 0.01	0.96±0.00
OPTPBE	1.09±0.00	1.85±0.00	1.66±0.01	1.95±0.01	1.89 ±0.01	0.96±0.00
OPTB88	1.09±0.00	1.85±0.00	1.66±0.01	1.95±0.01	1.88 ±0.01	0.96±0.00
**N = 34**						
PBE	1.09±0.00	1.85±0.00	1.67±0.00	1.95±0.01	1.88±0. 01	0.96±0.00
PBE-E	1.09±0.00	1.85±0.00	1.67±0.00	1.95±0.01	1.88± 0.01	0.96±0.00
PBE-D2	1.09±0.00	1.85±0.00	1.67±0.01	1.95±0.01	1.89 ±0.01	0.96±0.00
VDWDF	1.09±0.00	1.85±0.00	1.66±0.01	1.95±0.01	1.89± 0.01	0.96±0.00
OPTPBE	1.09±0.00	1.85±0.00	1.66±0.01	1.95±0.01	1.88 ±0.01	0.96±0.00
OPTB88	1.09±0.00	1.84±0.00	1.66±0.00	1.95±0.01	1.88 ±0.01	0.96±0.00
**N = 36**						
PBE	1.09±0.00	1.85±0.00	1.67±0.00	1.95±0.01	1.88±0. 01	0.96±0.00
PBE-E	1.09±0.00	1.85±0.00	1.67±0.00	1.95±0.01	1.88± 0.01	0.96±0.00
PBE-D2	1.09±0.00	1.85±0.00	1.67±0.01	1.95±0.01	1.89 ±0.01	0.96±0.00
VDWDF	1.09±0.00	1.85±0.00	1.67±0.01	1.95±0.01	1.88± 0.01	0.96±0.00
OPTPBE	1.09±0.00	1.85±0.00	1.67±0.01	1.95±0.01	1.88 ±0.01	0.96±0.00
OPTB88	1.09±0.00	1.85±0.00	1.67±0.01	1.95±0.01	1.88 ±0.01	0.96±0.00

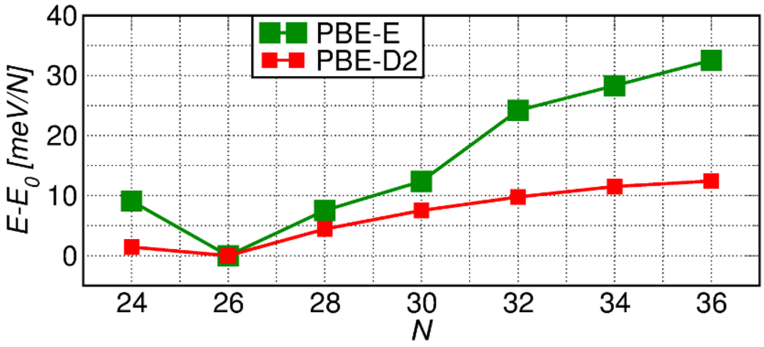

Figure S5. Relative dispersion energy normalized to the number of Al-atoms in the NT (*N*), and referenced to the computed minimum, for the PBE-D2 and PBE-E XC-functionals.

Table S3. Vacuum-aligned valence (VBEs) and conduction band edges (CBEs), and resulting band gaps (BGs)of the simulated NTs for increasing number of Al-atoms in the circumference (N) and the adopted XC-functionals.

N	PBE [eV]	PBE-E [eV]	PBE-D2 [eV]	VDWDF [eV]	OPTPBE [eV]	OPTB88 [eV]
**VBEs**						
**24**	-5.91	-5.91	-5.91	-6.17	-6.14	-6.15
**26**	-5.97	-5.97	-5.98	-6.14	-6.21	-6.17
**28**	-5.97	-5.97	-5.96	-6.16	-6.19	-6.21
**30**	-5.96	-5.91	-5.97	-6.14	-6.15	-6.21
**32**	-5.92	-5.90	-5.96	-6.13	-6.15	-6.21
**34**	-5.97	-5.96	-5.97	-6.18	-6.20	-6.24
**36**	-5.88	-5.89	-5.92	-6.11	-6.09	-6.14
**CBEs**						
**24**	-1.20	-1.20	-1.20	-1.69	-1.69	-1.52
**26**	-1.23	-1.23	-1.23	-1.70	-1.72	-1.49
**28**	-1.22	-1.22	-1.24	-1.70	-1.71	-1.55
**30**	-1.23	-1.19	-1.25	-1.70	-1.71	-1.55
**32**	-1.10	-1.19	-1.23	-1.70	-1.72	-1.55
**34**	-1.24	-1.24	-1.26	-1.71	-1.73	-1.56
**36**	-1.19	-1.19	-1.24	-1.69	-1.69	-1.52
**BGs**						
**24**	4.71	4.71	4.72	4.48	4.45	4.64
**26**	4.74	4.74	4.75	4.44	4.49	4.68
**28**	4.75	4.75	4.72	4.46	4.48	4.67
**30**	4.73	4.73	4.71	4.44	4.44	4.66
**32**	4.72	4.71	4.73	4.43	4.43	4.66
**34**	4.73	4.72	4.71	4.47	4.47	4.68
**36**	4.70	4.70	4.68	4.42	4.40	4.62

Table S4. PBE Vacuum-aligned valence (VBEs) and conduction band edges (CBEs), and resulting band gaps (BGs) for the minimum-energy $AlSi_N$-Me (N=28, 30) NTs optimized at VDWDF, OPTPBE and OPTB88 level. For ease of comparison the VDWDF, OPTPBE and OPTB88 values on the same geometries (from Table S3) have been reported within brackets.

N	XC-funtional for geometry opimization	VBEs [eV]	CBEs [eV]	BGs [eV]
**28**	**PBE**	-5.97	-1.22	4.75
**28**	**OPTB88**	-5.92 (-6.21)	-1.20 (-1.55)	4.72 (4.67)
**30**	**PBE**	-5.96	-1.23	4.73
**30**	**VDWDF**	-5.84 (-6.14)	-1.19 (-1.70)	4.66 (4.44)
**30**	**OPTPBE**	-5.89 (-6.15)	-1.22 (-1.71)	4.67 (4.44)
**30**	**OPTB88**	-5.91 (-6.21)	-1.21 (-1.55)	4.70 (4.66)

Table S5. Muliken charges (e) for the methyl groups (C_2,H_1) at the NT inner surface for each of the XC-functionals considered.

N	PBE [e]	PBE-E [e]	PBE-D2 [e]	VDWDF [e]	OPTPBE [e]	OPTB88 [e]
**24**	-0.3076	-0.3080	-0.3086	-0.3184	-0.3129	-0.3197
**26**	-0.3013	-0.3012	-0.3024	-0.3115	-0.3067	-0.3128
**28**	-0.2934	-0.2933	-0.2938	-0.3088	-0.2990	-0.3109
**30**	-0.2876	-0.2896	-0.2856	-0.3022	-0.2961	-0.2985
**32**	-0.2818	-0.2838	-0.2833	-0.2986	-0.2889	-0.2938
**34**	-0.2720	-0.2730	-0.2765	-0.2942	-0.2816	-0.2919
**36**	-0.2753	-0.2758	-0.2729	-0.2932	-0.2836	-0.2884

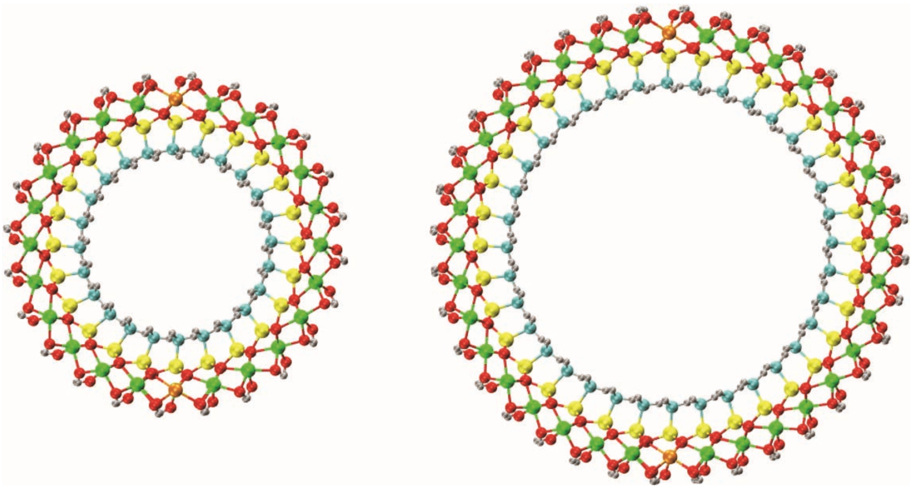

Figure S6. Front view of the (PBE) optimized $AlSi_{24}$-Me (Fe_2-$AlSi_{24}$-Me, left) and $AlSi_{36}$-Me (Fe_2-$AlSi_{36}$-Me, right) NTs with two (octahedral) Fe atoms substituted to two Al atoms. Same coloring scheme as in Figure 1, with the additional Fe atoms being colored orange.

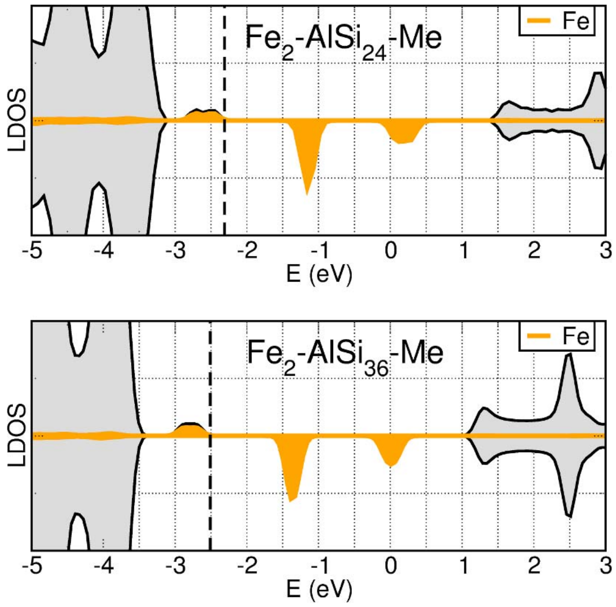

Figure S7. PBE total DOS (filled grey) and Fe-resolved LDOS (filled orange) plots for energy-favored ferromagnetic high-spin ordering of the Fe-doped NTs in Fig. S6. The dashed vertical line marks the position of the high-energy end of the occupied (L)DOS peak corresponding to Fe-dopant band gap states. At odds with results in Ref. [14f] for hydroxylated (not methylated) NTs with different XC-functionals (PW91, BLYP, B3LYP), high-spin (magnetic moment per Fe-atom: 3.7 μ_B) ferromagnetic ordering is computed to be favored by more than 1.3 eV and 1.6 eV eV over ferromagnetic low-spin (magnetic moment per Fe-atom: 1 μ_B) and anti-ferromagnetic (magnetic moment per Fe-atom: ±1 μ_B) solutions, respectively. The absence of details on whether different magnetic solutions were explored and converged in Ref. [14f] prevents further elaboration on these deviations.

Table S6. Computed surface dipole density (μ_σ), potential difference between inner and outer vacuum plateaus ($\Delta\overline{V}$), and electrostatically derived inner (R_{in}) and outer (R_{out}) NT radii for the considered models and XC-functionals. The PBE results for the pristine $AlSi_{24}$ NT are reported for comparison.

	μ_σ [pC m^{-1}]	$\Delta\overline{V}$ [eV]	R_{in} [Å]	R_{out} [Å]
**$AlSi_{24}$ (PBE)**	22.48	1.40	4.52	13.45
***N* = 24**				
PBE	14.24	0.84	4.17	13.57
PBE-E	14.24	0.84	4.17	13.57
PBE-D2	14.05	0.83	4.17	13.57
VdWDF	14.87	0.88	4.17	13.57
OPTPBE	15.10	0.89	4.17	13.57
OPTB88	15.34	0.92	4.17	13.56
***N* = 26**				
PBE	12.38	0.77	4.76	14.28
PBE-E	12.40	0.77	4.76	14.28
PBE-D2	12.14	0.75	4.76	14.28
VdWDF	14.75	0.92	4.88	14.40
OPTPBE	13.00	0.80	4.76	14.28
OPTB88	13.23	0.82	4.76	14.28
***N* = 28**				
PBE	11.88	0.78	5.59	15.11
PBE-E	11.92	0.78	5.59	15.11
PBE-D2	12.22	0.81	5.59	14.99
VdWDF	13.66	0.90	5.59	15.11
OPTPBE	12.76	0.84	5.59	15.11
OPTB88	12.87	0.85	5.59	15.11
***N* = 30**				
PBE	11.53	0.85	6.42	15.23
PBE-E	12.11	0.85	6.42	15.71
PBE-D2	11.49	0.80	6.42	15.82
VdWDF	13.32	0.92	6.43	15.94
OPTPBE	12.85	0.89	6.42	15.94
OPTB88	12.38	0.86	6.42	15.82
***N* = 32**				
PBE	11.65	0.85	7.38	16.66
PBE-E	11.88	0.87	7.37	16.66
PBE-D2	11.24	0.81	7.14	16.66
VdWDF	13.12	0.94	7.14	16.66
OPTPBE	12.55	0.90	7.14	16.66
OPTB88	12.16	0.87	7.14	16.66
***N* = 34**				
PBE	10.91	0.82	8.09	17.37
PBE-E	11.03	0.83	8.09	17.37
PBE-D2	10.74	0.81	8.09	17.37
VdWDF	11.93	0.89	8.09	17.49
OPTPBE	11.44	0.86	7.97	17.49
OPTB88	11.30	0.85	8.09	17.49
***N* = 36**				
PBE	11.66	0.89	8.67	18.18
PBE-E	11.62	0.89	8.67	18.18
PBE-D2	11.46	0.87	8.67	18.18
VdWDF	12.82	0.98	8.67	18.18
OPTPBE	12.73	0.97	8.67	18.18
OPTB88	12.28	0.94	8.67	18.18

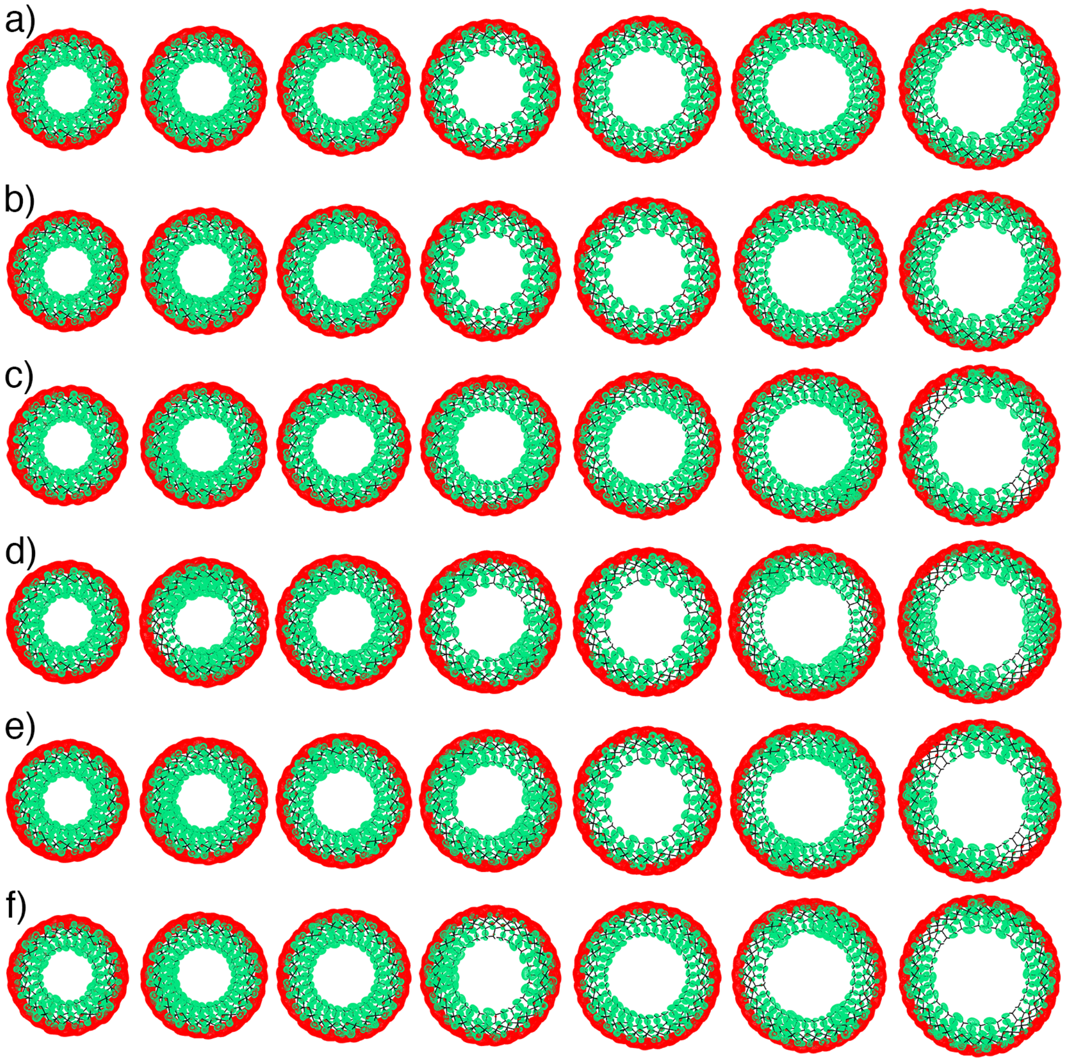

Figure S8. Real space separation of the VBE (green) and CBE (red) edges for the considered $AlSi_N$-Me NTs in the N=24 (left)-N=36 (right) range as a function of the XC-functional used. a) PBE, b) PBE-E, c) PBE-D2, d) VDWDF, e) OPTPBE, f) OPTB88. Regardless of the adopted XC-functional, the modelled VB-CB separation is qualitatively unaffected.

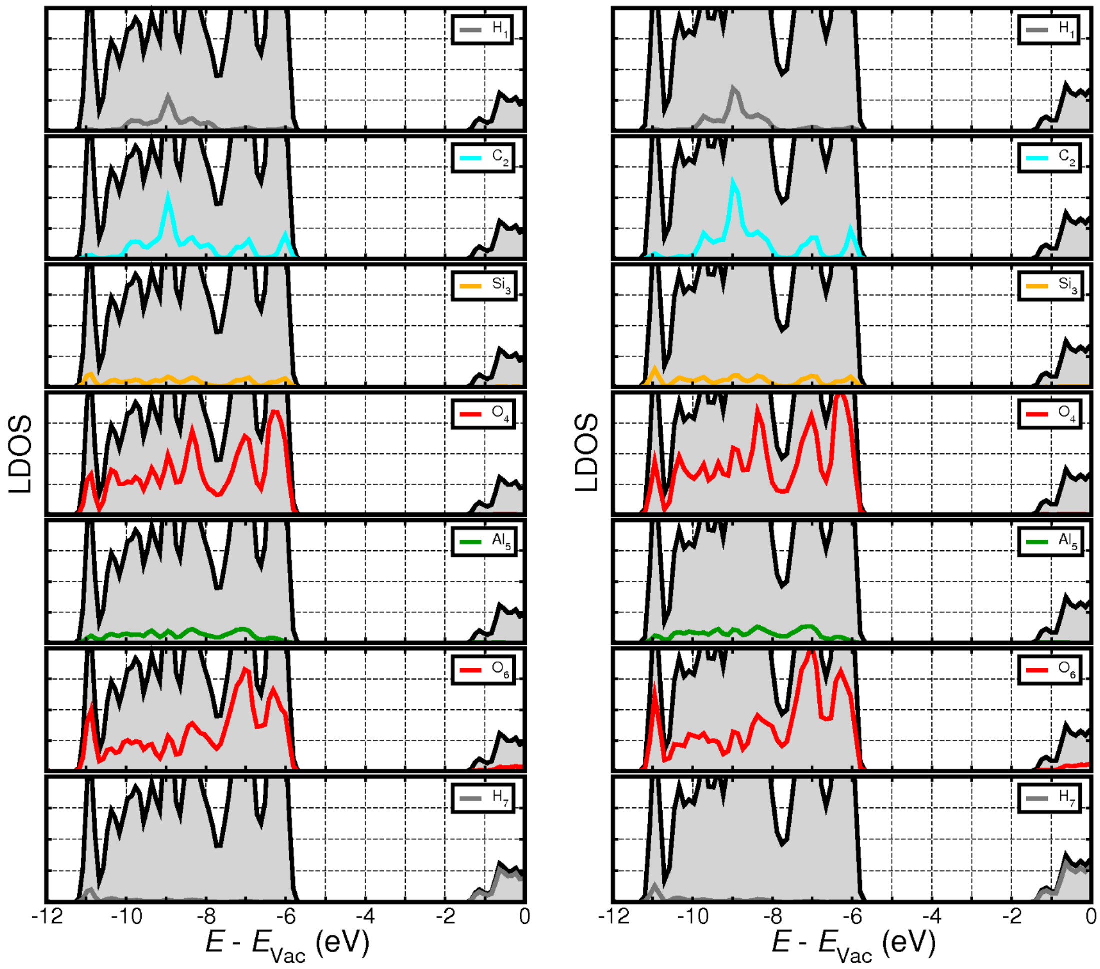

Figure S9. Vacuum-aligned **PBE** total DOS plot (filled grey) and layer resolved LDOS plots for the $AlSi_{28}$-Me (left) and $AlSi_{34}$-Me (right) NTs. See Figure 1a for the adopted layer-labeling.

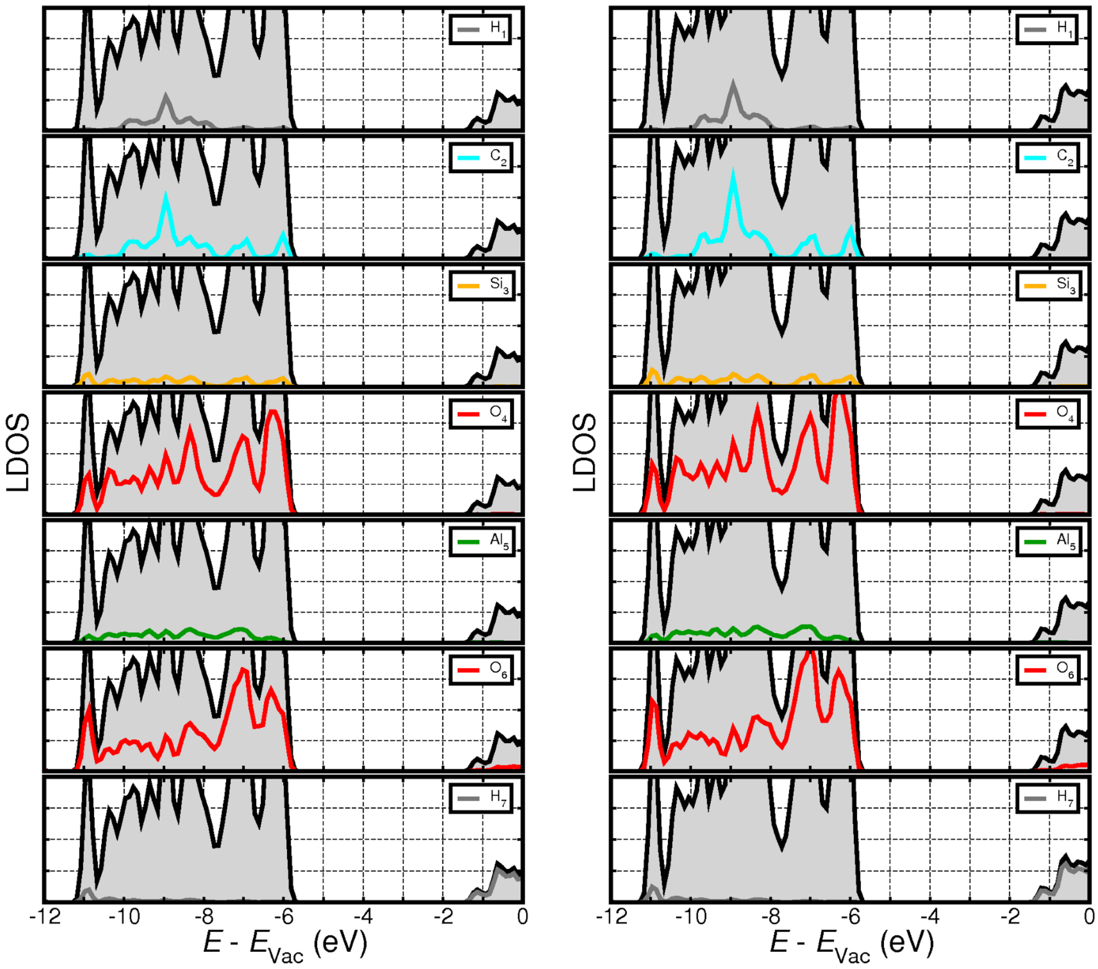

Figure S10. Vacuum-aligned **PBE-E** total DOS plot (filled grey) and layer resolved LDOS plots for the $AlSi_{28}$-Me (left) and $AlSi_{34}$-Me (right) NTs. See Figure 1a for the adopted layer-labeling.

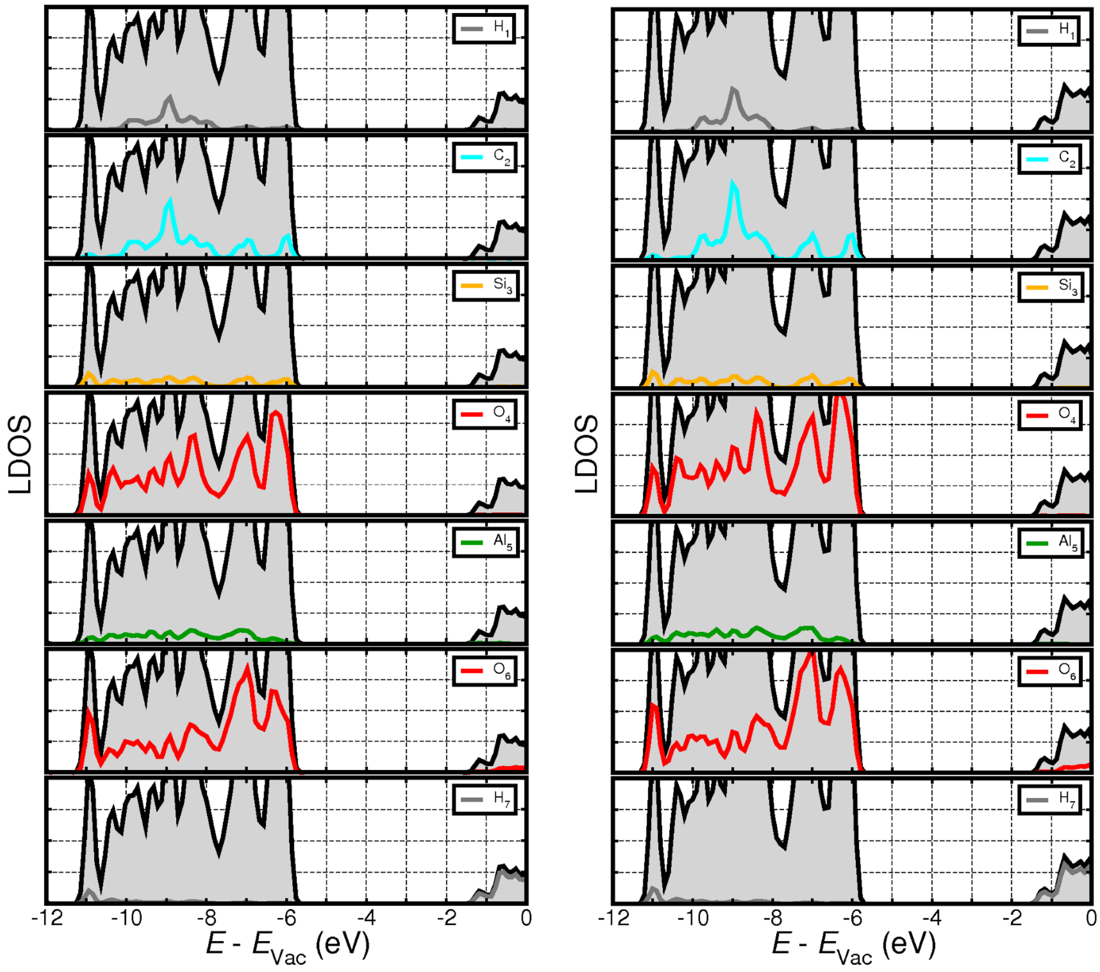

Figure S11. Vacuum-aligned **PBE-D2** total DOS plot (filled grey) and layer resolved LDOS plots for the $AlSi_{28}$-Me (left) and $AlSi_{34}$-Me (right) NTs. See Figure 1a for the adopted layer-labeling.

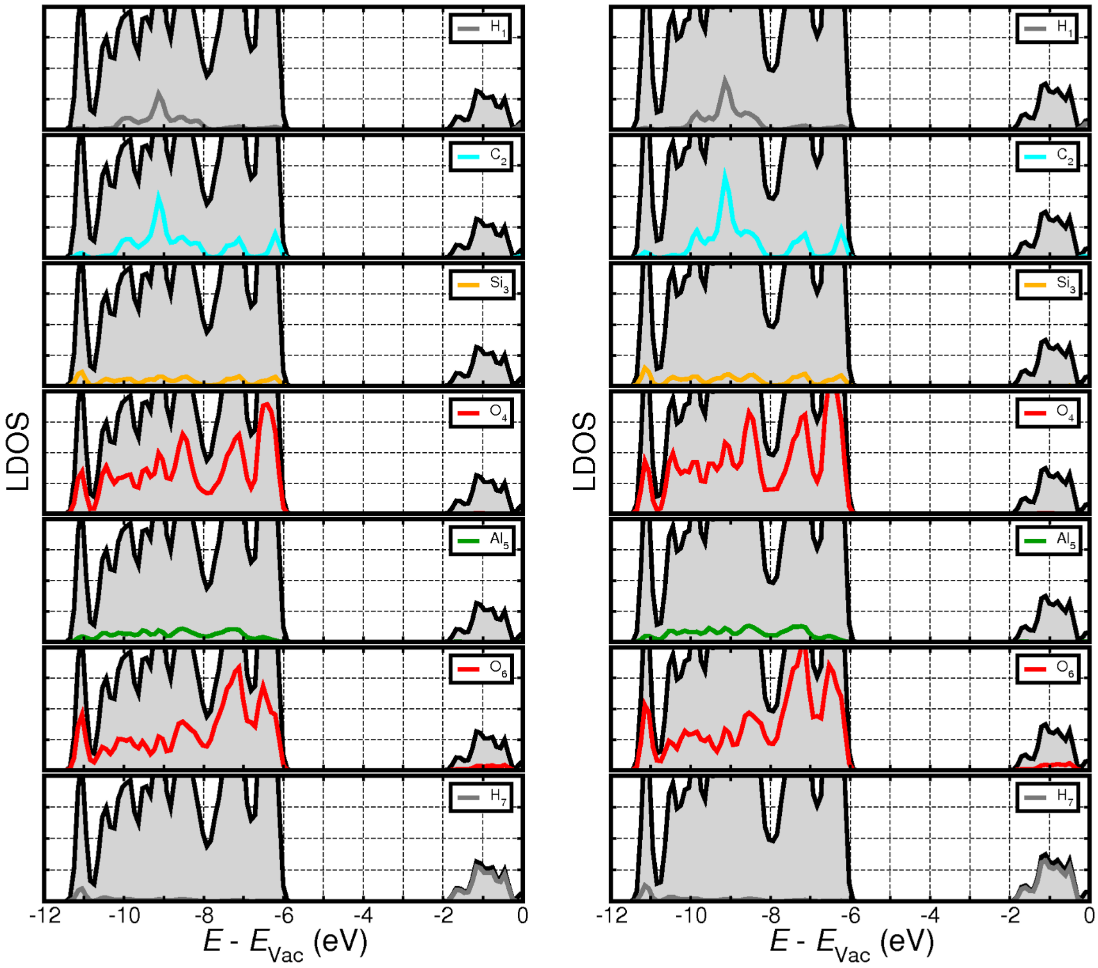

Figure S12. Vacuum-aligned **VDWDF** total DOS plot (filled grey) and layer resolved LDOS plots for the $AlSi_{28}$-Me (left) and $AlSi_{34}$-Me (right) NTs. See Figure 1a for the adopted layer-labeling.

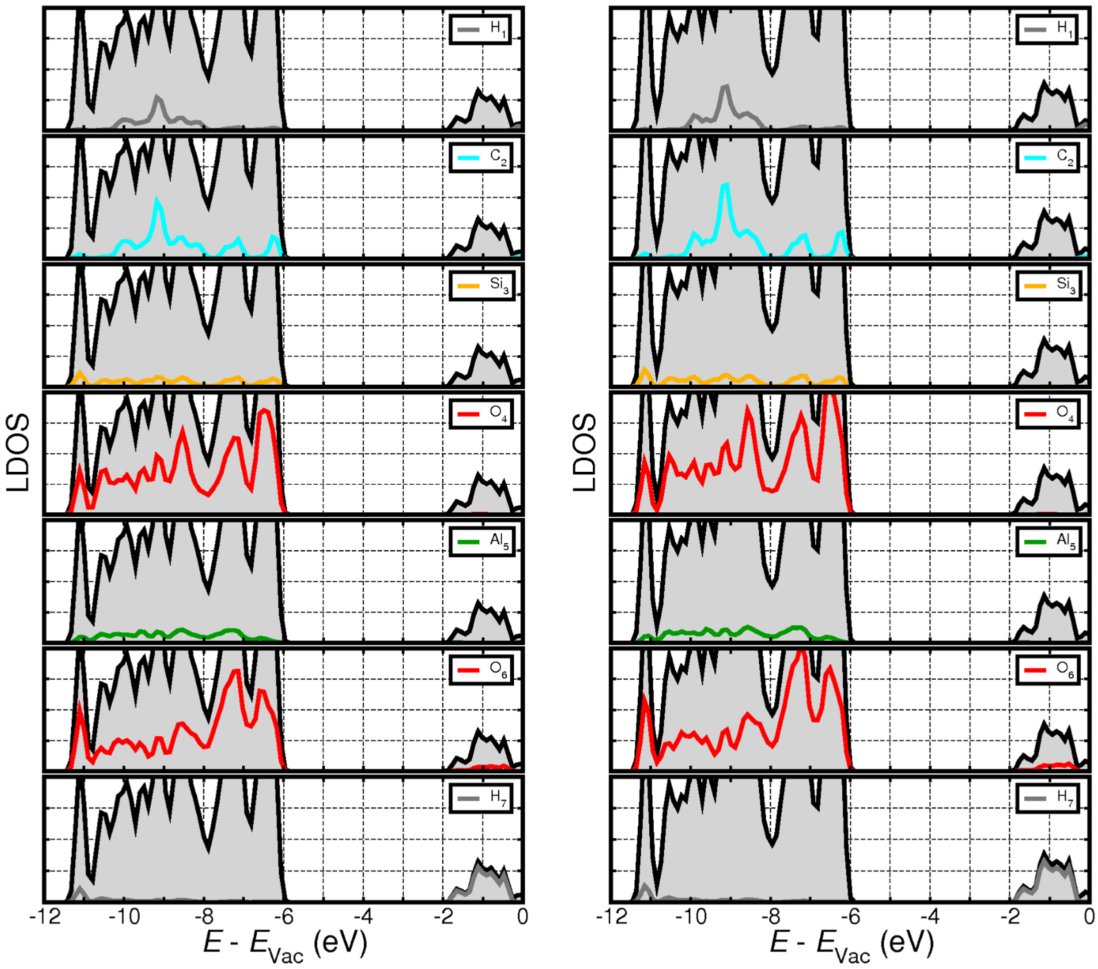

Figure S13. Vacuum-aligned **OPTPBE** total DOS plot (filled grey) and layer resolved LDOS plots for the $AlSi_{28}$-Me (left) and $AlSi_{34}$-Me (right) NTs. See Figure 1a for the adopted layer-labeling.

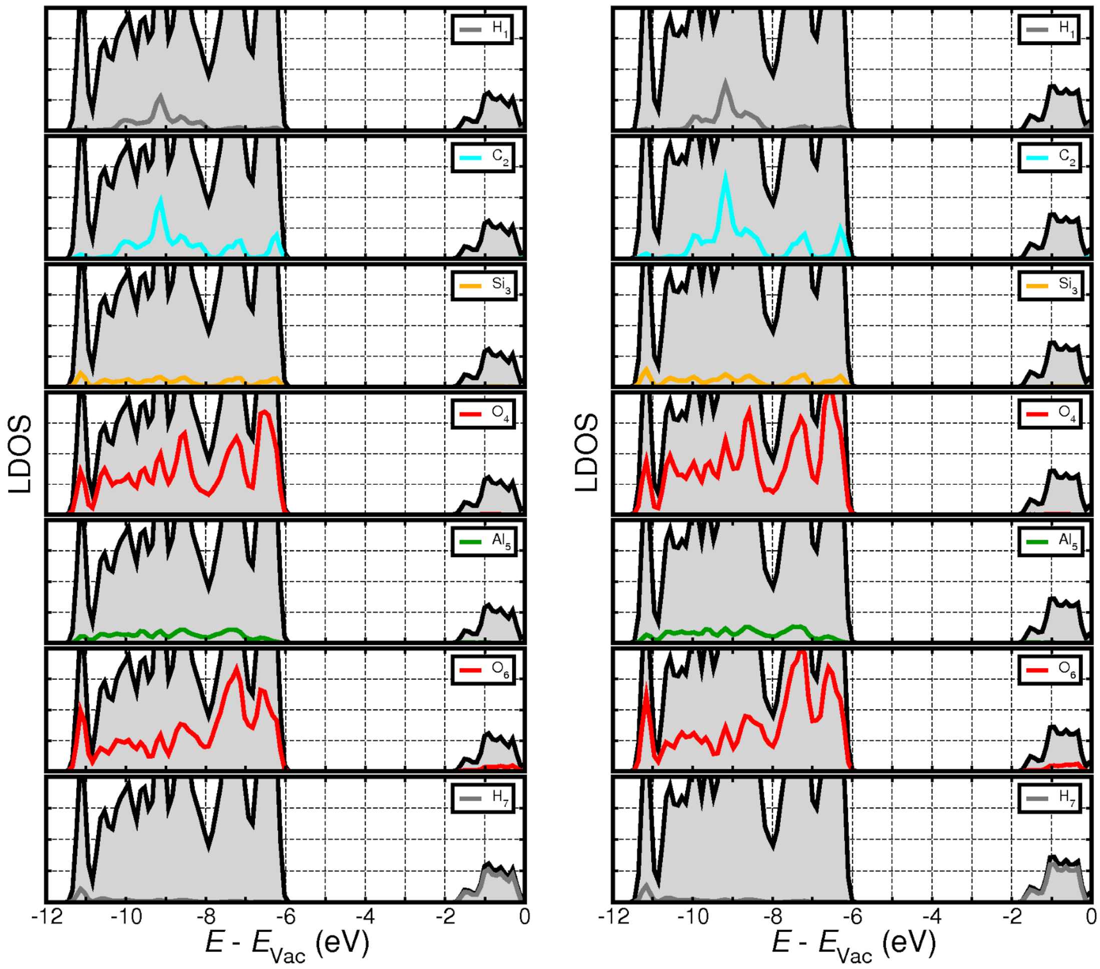

Figure S14. Vacuum-aligned **OPTB88** total DOS plot (filled grey) and layer resolved LDOS plots for the $AlSi_{28}$-Me (left) and $AlSi_{34}$-Me (right) NTs. See Figure 1a for the adopted layer-labeling.

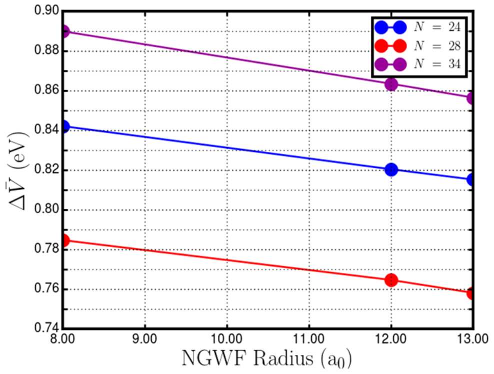

Figure S15. The calculated difference in the radially averaged electrostatic plateau inside and outside the NT cavity ($\Delta\overline{V}$) as a function of NGWF radius (bohr, a_0) for NTs containing 24, 28 and 36 Al-atoms within their circumference.

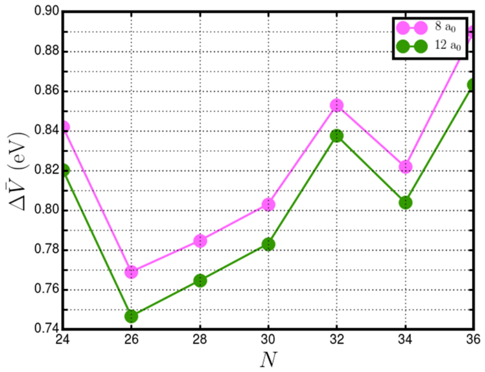

Figure S16. The calculated difference in the radially averaged electrostatic plateau inside and outside the NT cavity ($\Delta\overline{V}$) as a function of the number of Al-atoms within the tube circumference for 8 a_0 (purple) and 12 a_0 (green) NGWFs.

Table S7. The calculated potential difference between inner and outer vacuum plateau ($\Delta\overline{V}$), surface dipole density (μ_σ), and polarization (P) for $AlSi_{24}$-Me, $AlSi_{28}$-Me and $AlSi_{36}$-Me NTs as a function of the psinc basis set kinetic energy cutoff (E_c, in eV) and NGWFs radius (R, in Bohr, a_0).

System	E_c [eV]	R [Å]	$\Delta\overline{V}$ [eV]	μ_σ [pC m^{-1}]	P [C m^{-2}]
N=24					
	800	12	0.8186	14.006	0.01463
	1000	8	0.8438	14.267	0.01520
	1000	12	0.8204	13.870	0.01477
	1000	13	0.8152	13.782	0.01468
N=28					
	1000	8	0.7884	11.942	0.01256
	1000	12	0.7649	11.585	0.01219
	1000	13	0.7581	11.482	0.01208
	1500	8	0.7888	11.710	0.01274
N=36					
	1000	8	0.8866	11.614	0.0122
	1000	12	0.8635	11.311	0.0119
	1000	13	0.8564	11.220	0.0118

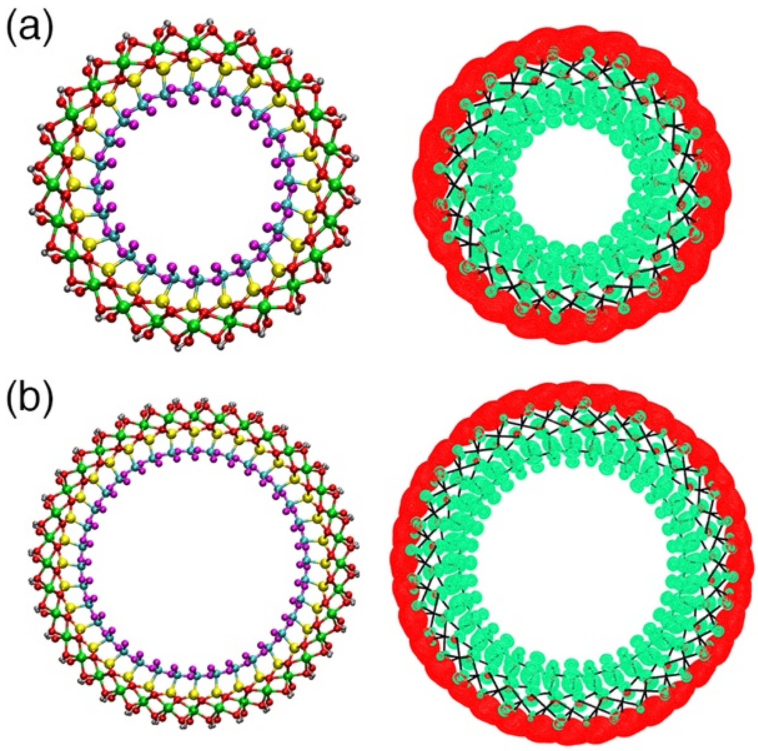

Figure S17. The PBE optimized geometry (left) and real-space separation (right) between the VBE (green) and CBE (red) of the (a) N=24 and (b) N=36 $AlSi_N$-CF_3 NTs. Same coloring scheme as in Figure 1, with the additional F-atoms being colored purple.

Table S8. The calculated surface dipole density (μ_σ), polarization (P), band gap (BG), vacuum aligned Valence Band (VBE) and Conduction Band (CBE) edges for the $AlSi_{24}$-CF_3 and $AlSi_{36}$-CF_3 NTs at PBE, PBE-D2 and VDWDF level.

System	μ_σ [pC m^{-1}]	P [C m^{-2}]	BG [eV]	VBE [eV]	CBE [eV^2]
**N=24**					
PBE	56.53	0.05862	4.104	-5.351	-1.247
PBE-D2	56.50	0.05859	4.104	-5.349	-1.245
VDWDF	57.60	0.05900	3.964	-5.768	-1.803
**N=36**					
PBE	44.52	0.04683	4.252	-5.501	-1.249
PBE-D2	44.53	0.04684	4.249	-5.492	-1.243
VDWDF	47.13	0.04837	4.062	-5.811	-1.749

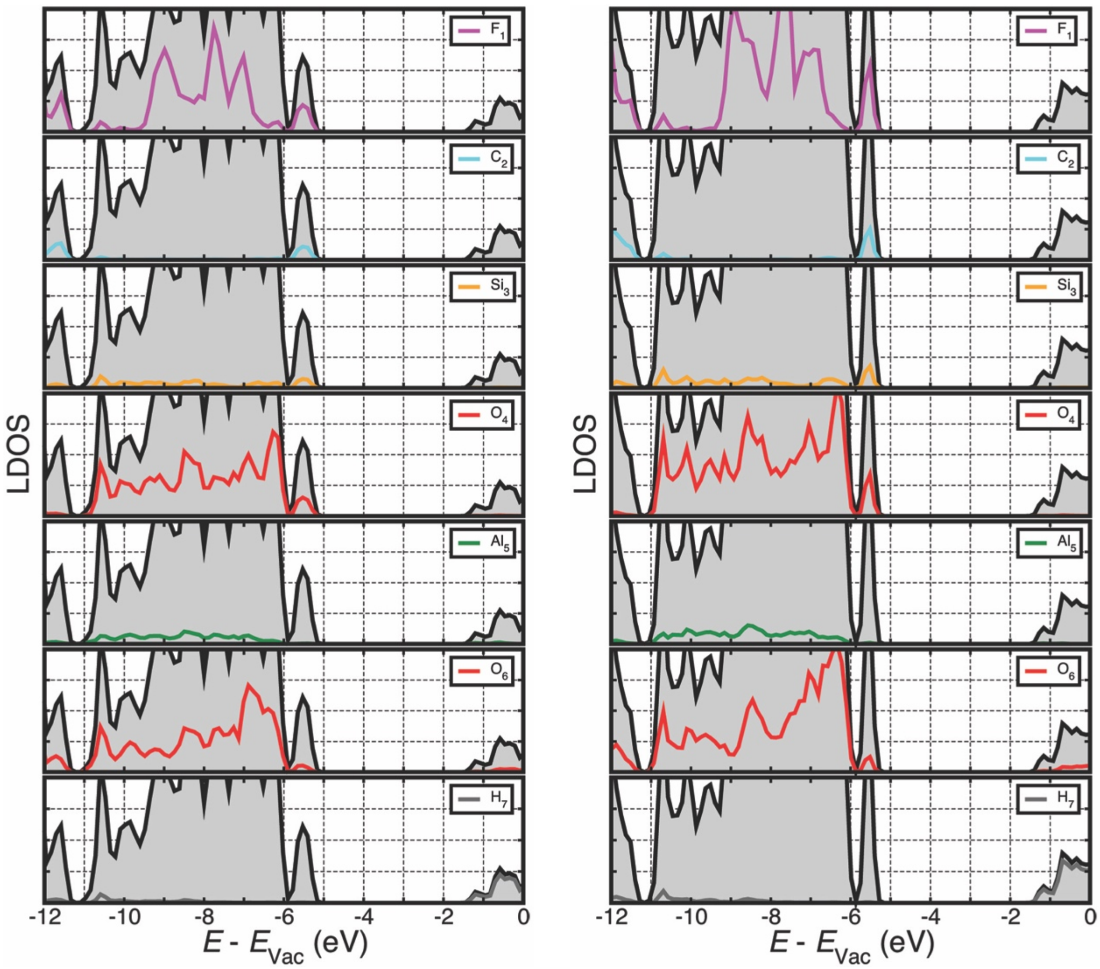

Figure S18. Vacuum-aligned **PBE** total DOS plot (filled grey) and layer resolved LDOS plots for the **$AlSi_{24}$-CF_3** (left) and **$AlSi_{36}$-CF_3** (right). See Figure 1a and S17 for the adopted layer-labeling.

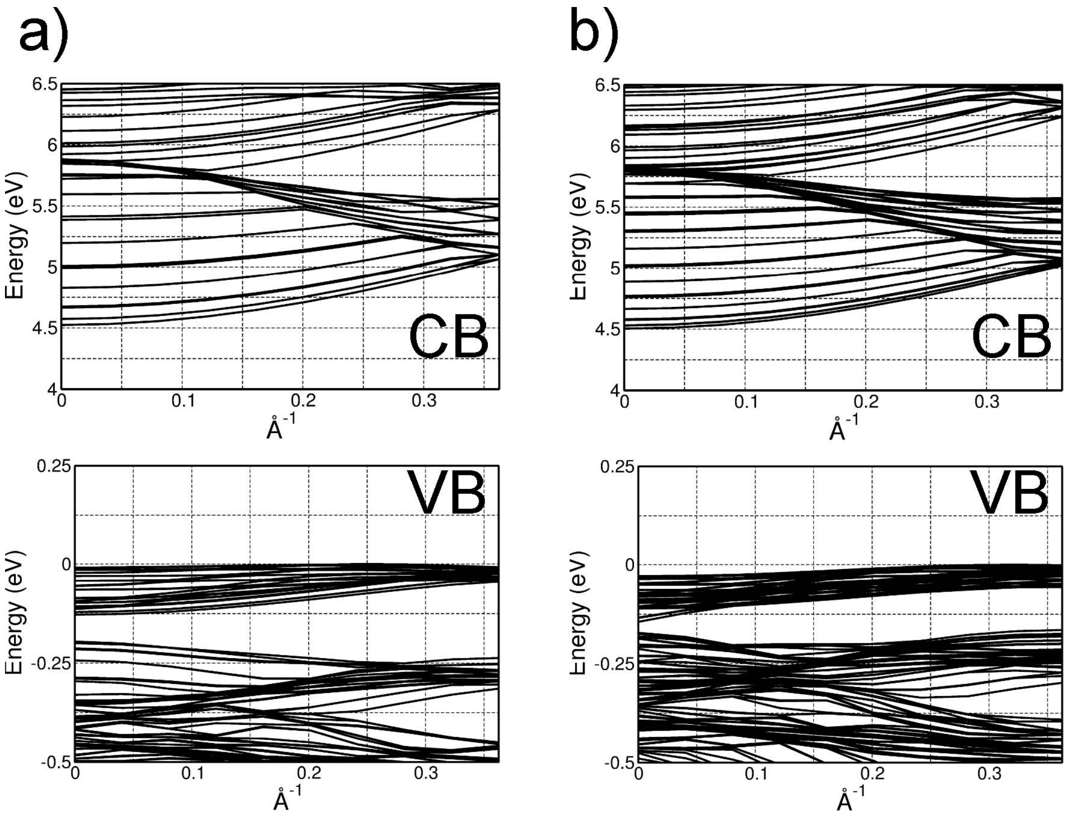

Figure S19. The calculated band structure along the NT-axis (ΓX direction, Ref. S7) for the methylated $AlSi_{24}$-Me (a) and $AlSi_{36}$-Me (b) NTs. VB: Valence Band, CB: Conduction Band. The energy scale has been referenced to the VB-maximum (0 eV). VB-maxima not at the centre of the Brilluoin zone (Γ-point) have previously been reported for other inorganic (ionic) nanotubes [S6].

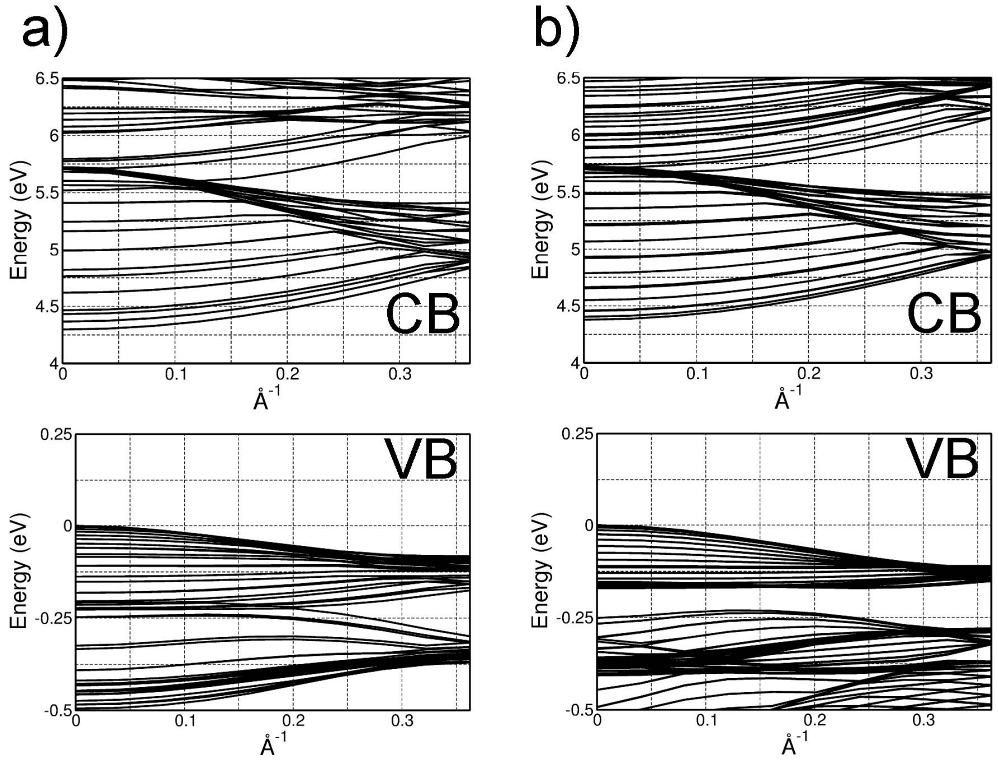

Figure S20. The calculated band structure along the NT-axis (ΓX direction, Ref. S7) for the pristine hydroxilated $AlSi_{24}$ (a) and $AlSi_{36}$ (b) NTs. VB: Valence Band, CB: Conduction Band. The energy scale has been referenced to the VB-maximum (0 eV). The computed results are in accordance with previously published PBE results for pristine (aluminosilicate) imogolite NTs [S7].

Table S9 The calculated effective electron (m_e) and hole (m_h) masses, in units of rest electron mass (m_0), for $AlSi_{24}$-Me and $AlSi_{36}$-Me NTs and corresponding hydroxylated analogs ($AlSi_{24/36}$). Results for α-Al_2O_3, γ-Al_2O_3 (from Ref. [S8]) are reported for comparison.

System	m_e/m_0	m_h/m_0
$AlSi_{24}$-Me	0.81	7.41
$AlSi_{36}$-Me	0.79	5.77
$AlSi_{24}$-Me	0.80	1.86
$AlSi_{36}$-Me	0.77	1.74
α-Al_2O_3	0.40 (Γ→A)<br>0.38 (⊥Γ→A)	7.5 (⊥Γ→A)<br>0.35 (Γ→A)
γ-Al_2O_3	0.40	1.3 (Γ→K)<br>>>1 (⊥Γ→K)

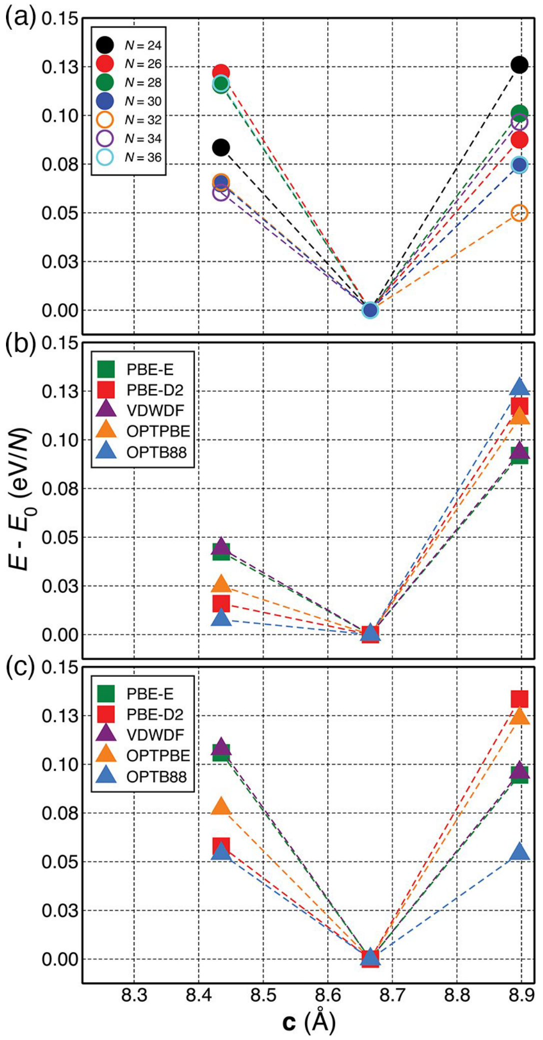

Figure S21. Relative DFT-energy, normalized to the number of Al-atoms in the NT (*N*) and referenced to the computed minimum, as a function of the NT-repeat unit length (*c*, see Fig. 1b) for the considered range of *N* and XC-functionals. *c* has been changed in multiple of the coarse FFT-grid separation [21] (0.231 Å) using the plane-wave optimized value from Ref. [11b] (c=8.666 Å) as starting point. a) the PBE energy minimum is computed for *c*=8.666 Å, regardless of *N* (i.e. of the NT radius). Regardless of the XC-functional used, *c*=8.666 Å is computed to yield an energy minimum also for the smallest $AlSi_{24}$-Me (b) and largest $AlSi_{36}$-Me (c) NTs, that bracket the whole range of considered *N*.

3. Supplementary References

[S1] J. D. Jackson, Classical Electrodynamics, John Wiley & Sons, Inc.

[S2] J. Junquera, M. H Cohen, K. M. Rabe, *J. Phys. Condens. Matt.* **19**, 213203 (2007).

[S3] C.-K. Skylaris, P. D. Haynes, A. A. Mostofi, M. C. Payne, *J. Chem. Phys.* **122** 084119 (2005).

[S4] G. Kresse, J. Furthmüller, *Phys. Rev. B* **54** 11169 (1996).

[S5] J.P. Perdew, K. Burke, M. Ernzerhof, *Phys. Rev. Lett.* **77** 3865 (1996).

[S6] J. Xiao, M. Long, X. Li., H. Xu, H. Huang, Y. Gao, *Sci. Rep.* **4**, 4327 (2014).

[S7] M. Zhao, Y. Xia, L. Mei, *J. Phys. Chem. C* **113**, 14834 (2009).

[S8] T. V Perevalov, A. V. Shaposhnikov, V. A. Gritsenko, Microelectron. Eng. **86**, 1915 (2009).
